# Transmembrane stem cell factor protein therapeutics enhance revascularization in ischemia without mast cell activation

**DOI:** 10.1038/s41467-022-30103-2

**Published:** 2022-05-06

**Authors:** Eri Takematsu, Miles Massidda, Jeff Auster, Po-Chih Chen, ByungGee Im, Sanjana Srinath, Sophia Canga, Aditya Singh, Marjan Majid, Michael Sherman, Andrew Dunn, Annette Graham, Patricia Martin, Aaron B. Baker

**Affiliations:** 1 Department of Biomedical Engineering, University of Texas at Austin, Austin, TX USA; 2 Department of Biochemistry & Molecular Biology, University of Texas Medical Branch, Galveston, TX USA; 3 Department of Biological and Biomedical Sciences, School of Health and Life Sciences, Glasgow Caledonian University, G4 0BA Scotland, UK; 4 Institute for Cellular and Molecular Biology, University of Texas at Austin, Austin, TX USA; 5 The Institute for Computational Engineering and Sciences, University of Texas at Austin, Austin, TX USA; 6 Institute for Biomaterials, Drug Delivery and Regenerative Medicine, University of Texas at Austin, Austin, TX USA

**Keywords:** Angiogenesis, Biomedical engineering, Drug discovery

## Abstract

Stem cell factor (SCF) is a cytokine that regulates hematopoiesis and other biological processes. While clinical treatments using SCF would be highly beneficial, these have been limited by toxicity related to mast cell activation. Transmembrane SCF (tmSCF) has differential activity from soluble SCF and has not been explored as a therapeutic agent. We created novel therapeutics using tmSCF embedded in proteoliposomes or lipid nanodiscs. Mouse models of anaphylaxis and ischemia revealed the tmSCF-based therapies did not activate mast cells and improved the revascularization in the ischemic hind limb. Proteoliposomal tmSCF preferentially acted on endothelial cells to induce angiogenesis while tmSCF nanodiscs had greater activity in inducing stem cell mobilization and recruitment to the site of injury. The type of lipid nanocarrier used altered the relative cellular uptake pathways and signaling in a cell type dependent manner. Overall, we found that tmSCF-based therapies can provide therapeutic benefits without off target effects.

## Abstract (web summary)

Stem cell factor (SCF) is a cytokine with great potential for treating ischemia, stroke and heart attack, but therapies using SCF have been limited by acute allergic responses. The work describes a new therapeutic using transmembrane SCF delivered in lipid nanocarriers that is effective in treating ischemia without off-target effects.

## Introduction

Over the past three decades, protein therapeutics have emerged as a powerful approach to drug development^1,2^. The first use of a therapeutic protein developed was insulin, used as a therapy for diabetes mellitus^3^. Since then, over 200 protein-based compounds have been approved for clinical use and over 250 proteins are currently in various stages of clinical evaluation^4,5^. While proteins have immense potential in bridging the gap between a scientific discovery and translation into clinical therapy, there remain major limitations and challenges in their delivery and efficacy^6^. This has been particularly the case with membrane proteins as these molecules are natively found in the complex lipid bilayer of the cell membrane and often require this environment for proper function and solubility^7^. Virtually all disease processes involve membrane proteins as receptors, co-receptors, or membrane bound factors that are needed to transmit cellular signals^8–10^. Thus, the delivery of membrane protein therapeutics may provide a rich strategy for enabling next generation of protein therapeutics.

Stem cell factor (SCF) is a hematopoietic cytokine that signals through the c-Kit receptor (CD117)^11^, and is also known as Kit ligand, Steel factor or mast cell growth factor. Through alternative splicing SCF is expressed in cells initially as a transmembrane protein that subsequently enzymatically cleaved into soluble SCF or a shorter isoform that lacks the cleavable domain and remains membrane bound as transmembrane SCF (tmSCF)^12^. Signaling through SCF induced c-Kit activation is key to the maintenance of the hematopoietic stem cells (HSCs) and progenitor cells in the bone marrow^13,14^. There are several potential uses for SCF in therapeutic applications including improving the survival and expansion of HSCs following exposure to radiation^15,16^, inducing neuroprotective effects following stroke^17–19^, and enhancing the recovery of the heart following myocardial infarction. However, SCF also has an important role in regulating mast cell maturation and activation^20,21^. Treatment with exogenous SCF leads to mast cell activation and anaphylaxis in animal studies and clinical trials, severely limiting its therapeutic application^22–29^.

In the body, SCF is expressed both as a longer isoform that is initially a transmembrane protein but then released as soluble SCF through enzymatic cleavage and as a shorter isoform that remains as transmembrane SCF^30^. Transmembrane SCF is found in the stromal cells of the bone marrow where it functions to support the proliferation and survival of progenitor cells^31^. Soluble and transmembrane SCF differ in terms of their ability to activate the c-Kit receptor, induce cellular responses, and ability to promote adhesion between hematopoietic stem cells and extracellular matrix^11,32^. Several reports have further demonstrated that tmSCF can induce prolonged activation of the c-Kit receptor^33^ and longer term proliferation of CD34^+^ hematopoietic cells in comparison to soluble SCF^34^. While studies have supported that gene expression of tmSCF can improve recovery following myocardial infarction^22^, delivery of exogenous tmSCF has not been explored as therapeutic strategy in preclinical or clinical studies.

In this study, we developed therapeutics based on tmSCF delivered in lipid nanodiscs or as proteoliposomes. Our studies demonstrated that tmSCF-based therapies do not induce mast cell activation in mice, in contrast to soluble SCF. Further, we found that tmSCF nanodiscs and proteoliposomes enhanced the recovery from hind limb ischemia of both wild type and ob/ob mice. Trafficking studies demonstrated that mast cells mainly use clathrin-mediated pathways to uptake SCF, while they use both clathrin- and caveolin-mediated pathways to internalize tmSCF therapies. In addition, the type of nanocarrier used altered the signaling, internalization pathways, and relative activity towards in endothelial cells or endothelial progenitor cells (EPCs). Overall, our studies suggest that therapeutics based on tmSCF can enhance stem cell recruitment and revascularization without toxic activities including mast cell activation.

## Results

### Synthesis of transmembrane stem cell factor nanodiscs and proteoliposomes

**Fig. 1:**
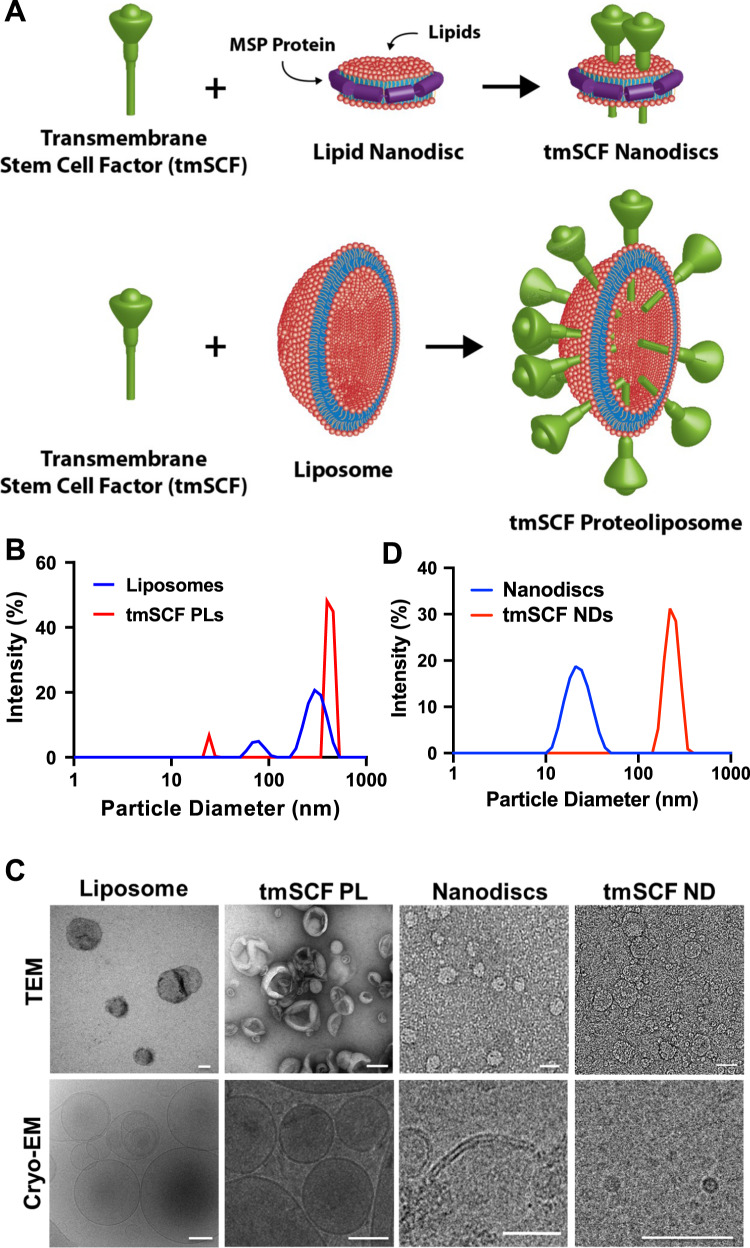
Characterization of tmSCFPLs and tmSCFNDs by DLS and electron microscopy. **A** Schematic illustration of tmSCF proteoliposomes (tmSCFPLs) and tmSCF nanodiscs (tmSCFNDs). **B** Size distribution for liposomes and proteoliposome with tmSCF measured by dynamic light scattering. **C** Size distribution for nanodiscs and tmSCF nanodiscs. **D** Representative TEM and cryo-EM images of liposomes, tmSCFPLs, nanodiscs, and tmSCFNDs. Scale bar = 100 nm.

We created several formulations of recombinant tmSCF to test the effectiveness and differential response in comparison to soluble SCF. Our previous studies had identified that embedding transmembrane proteins in proteoliposomes can alter their therapeutic and signaling properties^35,36^. In addition, recent work has identified methods for creating lipid nanodiscs that are stabilized by membrane scaffolding proteins (MSPs). Thus, we aim to create two types of nanocarriers, tmSCF proteoliposomes, and nanodiscs to deliver tmSCF effectively to ischemic sites (Fig. 1A). Transmembrane stem cell factor (tmSCF) protein was first harvested and purified as described in “Methods” section. SDS-PAGE, western blotting, and silver staining were performed to analyze the purity of the final concentrated samples. The results of silver staining and western blotting confirmed the purity of tmSCF protein (Supplementary Fig. 1). We measured the size of the purified tmSCF and found that there is likely self-association/aggregation in the absence of a lipid carrier (Supplementary Fig. 2). We fabricated tmSCF proteoliposomes (tmSCFPLs) using detergent depletion as previously described^35,36^. The initial liposomes were around 350 nm in diameter as expected from the extrusion membrane used (Fig. 1B). After embedding tmSCF, the size was increased to 450 nm. We further verified the liposomal structure using TEM and cryo-EM (Fig. 1C). Another type of nanocarrier is nanodiscs made by 1-palmitoyl-2-oleoyl-sn-glycero-3-phosphocholine (POPC) lipid and Membrane Scaffold Protein 1D1 (MSP1D1). MSP1D1 comes together to form a “lariat” structure that stabilizes the hydrophobic edges of a lipid nanodisc. Purified nandiscs had a size of 20–30 nm and following tmSCF embedding and detergent depletion, the size increased to 150 nm (Fig. 1D). The structure of the nanodiscs was further verified using TEM and cryo-EM, which revealed nanodisc structures (Fig. 1C). Zeta potential of tmSCF, tmSCFPLs, and tmSCFNDs were −4.37, −6.78, and −4.85 mV respectively. (Supplementary Table 1).

### Transmembrane stem cell factor-based treatments do not induce mast cell activation

**Fig. 2:**
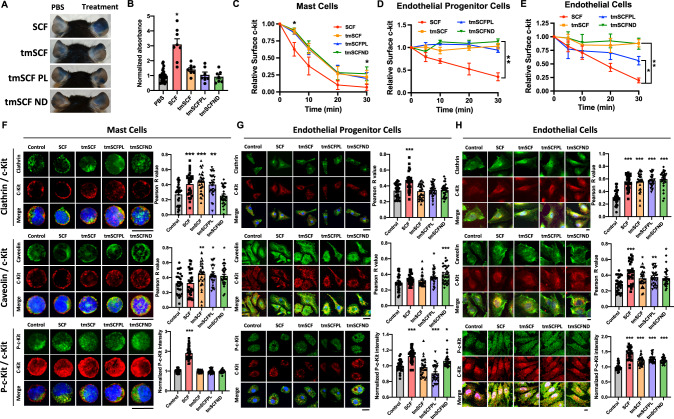
Nanocarriers alter the uptake mechanism of tmSCF-based therapeutics. **A** Representative pictures of mice ears after Evan’s blue extravasation assay. PBS was injected to the left ear as a control and treatments were injected to the right ear (*n* = 7 biologically independent mice examined over two independent experiments). **B** Quantification result of the absorbance at 620 nm. *p < 0.001 vs control. Two-sided one-way ANOVA with Turkey correction was used. (*n* = 7 for tmSCFPL and tmSCFND, 8 for SCF and tmSCF, 26 for PBS). **C** Surface staining for c-Kit on MC9 mast cells was monitored by flow cytometry. The intensity was normalized to 0 min time point to evaluate the treatment uptake kinetics (*n* = 6). *p = 0.0275 at 5 min, *p* = 0.048 at 30 min on tmSCFND vs SCF. Kruskal–Wallis test with Dunn’s post hoc was used. **D** Surface c-Kit on bone marrow mononuclear cells monitored by flow cytometry (*n* = 4 for alginate, 5 = SCF, tmSCFND, and 6 = tmSCFPL). **p = 0.0028 on tmSCFND vs SCF. Kruskal–Wallis test with Dunn’s post hoc was used. **E** Surface c-Kit on HUVECs were monitored by flow cytometry (*n* = 8 for time point 30 min, *n* = 4 for other time points). *p = 0.0138 on tmSCFPL vs SCF and **p < 0.001 on tmSCFND and tmSCF versus SCF. Two-sided one-way ANOVA with Dunnett’s post hoc test was used. **F** (Top) Representative pictures of single mast cell stained with clathrin and c-Kit. Pearson’s *R* value of the colocalization of clathrin and c-Kit (*n* = 30). *p* = 0.0009 on SCF vs control, *p* < 0.0001 on tmSCF vs control, and *p* = 0.0017 tmSCPL vs control. (Middle) Representative pictures of single mast cell stained with caveolin and c-Kit. Pearson’s *R* value of the colocalization of caveolin and c-Kit (*n* = 30). *p* = 0.0053 on tmSCF vs control, *p* = 0.0254 on tmSCPL vs control, and *p* = 0.0382 on tmSCFND vs control. (Bottom) Representative pictures of single mast cell stained with c-Kit and p-C-kit. The p-c-Kit mean intensity inside of mast cells was quantified (*n* = 30). *P* < 0.0001 on SCF vs control. Two-sided one-way ANOVA with Dunnett’s post hoc test was used. **G** (Top) Representative pictures of EPCs stained with clathrin and c-Kit. Pearson’s *R* value of the colocalization of clathrin and c-Kit (*n* = 30). *p* < 0.0001 on SCF vs control. (Middle) Representative pictures of EPCs stained with caveolin and c-Kit. Pearson’s *R* value of the colocalization of caveolin and c-Kit (*n* = 30). *p* = 0.0117 on tmSCFPL vs control and *p* < 0.0001 on tmSCND vs control. (Bottom) Representative pictures of EPCs stained with c-Kit and p-c-Kit. The p-c-Kit mean intensity inside of EPCs was quantified (*n* = 30). *p* < 0.0001 on SCF vs control, p = 0.0004 on tmSCPL vs control, and *p* = 0.0304 on tmSCFND vs control. Two-sided one way ANOVA with Dunnett’s post hoc test was used. **H** (Top) Representative pictures of HUVECs stained with clathrin and c-Kit. Pearson’s *R* value of the colocalization of clathrin and c-Kit (*n* = 30). *p* < 0.0001 on all treatment group vs control. (Middle) Representative pictures of HUVECs stained with caveolin and c-Kit. Pearson’s *R* value of the colocalization of caveolin and c-Kit (*n* = 30). *p* < 0.0001 on SCF vs control, *p* = 0.0361 on tmSCF vs control, *p* = 0.0062 on tmSCFPL vs control, and *p* = 0.0278 on tmSCFND vs control. (Bottom) Representative pictures of HUVECs stained with c-Kit and p-c-Kit. The p-c-Kit mean intensity inside of HUVECs was quantified (*n* = 30). *p* < 0.0001 on all treatment group vs control. Two-sided one-way ANOVA with Dunnett’s post hoc test was used. Scale bar is 30 µm. * indicates *p* value < 0.05, ** < 0.01, and *** <0.001. All the error bars are SEM.

A major limitation in the clinical use of soluble SCF is the activation of mast cells and potential induction of anaphylaxis^21,37^. To test whether tmSCF activates mast cells, we injected mice with Evan’s blue dye and then locally injected PBS, soluble SCF, tmSCF, tmSCFPLs, or tmSCFNDs into the ears of the mice. After three hours, we imaged the ears and extracted the dye with DMSO to quantify the vascular leakage induced by the treatments. We found that SCF induced vascular leakage but tmSCF-based therapies did not induce this response (Fig. 2A, B). Another indication of mast cell activation is the changes in body temperature. We injected treatments intravenously and monitored the mice body temperature changes over time. We observed a significant body temperature drop in SCF group while the body temperature changes in other treatment groups were similar to control-treated animals (Supplementary Fig. 3). We further conducted a toxicology test to examine any toxic effects of our treatments. We intravenously injected 400 µg/kg of each treatment and monitored the changes in body weight, body temperature, and appearance every day for one week (Supplementary Fig. 4A, B). There were no significant changes in body weight and body temperature, but we observed an occasional skin irritation in SCF treated group. In addition, histological analysis of the mice revealed some edema in the skin of SCF-treated mice but not mice given the other treatments (Supplementary Fig. 4C). There were no morphological changes in the liver or kidney of the mice with any of the treatments in comparison to saline-treated mice (Supplementary Fig. 4D).

### Transmembrane stem cell factor-based treatments cause slower c-Kit internalization

SCF interacts with its cognate receptor c-Kit leading to dimerization, signaling, and internalization through either a caveolin or clathrin-mediated pathway^38^. We measured the surface c-Kit internalization by flow cytometry and found that tmSCF-based treatments had slower uptake in mast cells (MC9 cells), bone marrow mononuclear cells (BMMNCs), and endothelial cells in comparison to soluble SCF (Fig. 2C–E). In endothelial cells, the internalization of c-Kit was the fastest for the tmSCFPLs in comparison to the other tmSCF formulations but still significantly slower than soluble SCF (Fig. 2E). Soluble SCF can be internalized by mast cells using a caveolin- or clathrin-mediated pathway^39^. The caveolin-mediated pathway has slower uptake kinetics and leads to reduced proliferation and migration of mast cell^39^. The clathrin-mediated pathway has faster uptake kinetics and leads to increased proliferation and migration of mast cells^39^. To examine the uptake mechanism of the tmSCF formulation, we treated cells with SCF and the tmSCF-based treatments and then performed immunostaining for c-Kit and clathrin or caveolin followed by an analysis for co-localization between the markers. We found that mast cells preferentially use clathrin-mediated pathways to internalize SCF and caveolin-mediated pathways to internalize tmSCFNDs (Fig. 2F). Mast cells use both clathrin- and caveolin-mediated pathway to uptake tmSCF or tmSCFPLs. These results correspond well with the protein uptake kinetics results, showing faster uptake of the SCF therapy (a hallmark of clathrin-mediated uptake) and slower uptake of the tmSCF-based therapies (a hallmark of caveolin-mediated uptake; Fig. 2C)^39^. Next, we conducted the same experiment on bone marrow-derived EPCs and found that they preferentially use clathrin-mediated pathways to uptake SCF while they use a caveolin-mediated pathway to uptake tmSCFPLs and tmSCFNDs (Fig. 2G). We found that endothelial cells use both clathrin- and caveolin-mediated pathways to uptake SCF, tmSCF, tmSCFPLs, and tmSCFNDs (Fig. 2H).

### Transmembrane SCF-based therapies activate the c-Kit pathway in endothelial cells and EPCs but not mast cells

In MC9 mast cells, SCF treatment induced significantly more phosphorylation of c-Kit than tmSCF-based treatments (Fig. 2F). In EPCs, SCF and tmSCFND treatment induced significantly more phosphorylation of c-Kit in comparison to control, indicating that SCF and tmSCFNDs may have angiogenic activity related to the SCF/c-Kit pathway (Fig. 2G). In endothelial cells, all treatments induced significantly more phosphorylation of c-Kit in comparison to control (Fig. 2H). These results support that there is lower pleotropic activity from tmSCF treatments on mast cells. The type of formulation used for delivering tmSCF tailors its trophic specificity, with preferential activation and faster internalization of tmSCFPLs by matured endothelial cells and greater activity towards EPCs using tmSCFNDs.

### Transmembrane SCF proteoliposomes induce greater endothelial cell tube formation in comparison to SCF or tmSCF nanodiscs

To measure the angiogenic activity of tmSCF based therapies, we performed a tube formation assay using endothelial cells. While tmSCF protein itself induced limited tube formation, tmSCFPLs had 16 to 18 fold greater activity (Supplementary Fig. 5A, B). In contrast, we did not see a significant induction of tube formation with tmSCFND treatment (Supplementary Fig. 5C, D). We also tested endothelial cell migration and proliferation but did not see any significant differences for any of our treatments compared to the control (Supplementary Fig. 6).

### Transmembrane stem cell factor proteoliposomes and nanodiscs enhance revascularization in wild type mice

**Fig. 3:**
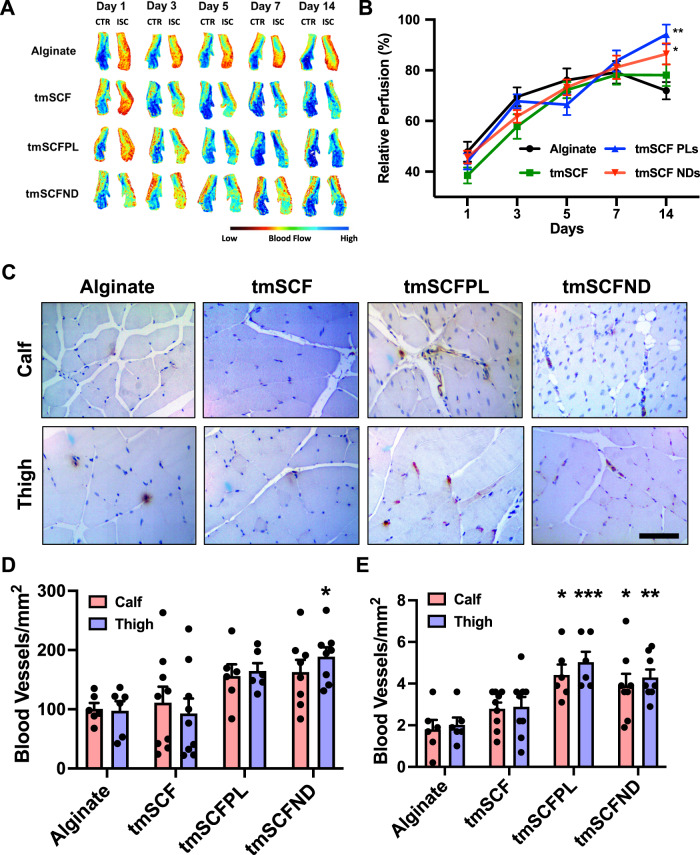
Evaluation of treatments in a hindlimb ischemia model on wild type mice. **A** Representative mice foot images taken by laser speckle imaging. **B** Relative blood flow recovery after hind limb ischemia surgery on WT mice (*n* = 13 for tmSCFND, 12 for other groups). *p* = 0.006 (tmSCFPL vs control) and *p* = 0.037 (tmSCFND vs control) * indicates *p* value < 0.05 **p < 0.01. **C** Immunostaining for PECAM on mice on the muscles from the ischemic calf and thigh muscle of the mice after 14 days. Scale bar = 100 µm. **D** Quantification small blood vessels in the calf and thigh muscle counted from PECAM immunostaining images (*n* = 7 for tmSCF, 4 for other groups). *p = 0.0116 on tmSCFND vs alginate. **E** The number of large blood vessels in the tissues (*n* = 7 for tmSCF, 4 for other groups). *p* = 0.043 on tmSCFPL vs control and *p* = 0.0295 on tmSCND vs control on calf muscle. *p* = 0.0005 on tmSCFPL vs control and *p* = 0.0038 on tmSCFND vs control on thigh muscle. Two-sided one-way ANOVA with Dunnett’s post hoc test was used. * indicates *p* value < 0.05, ** < 0.01, and *** <0.001. All the error bars are SEM.

To deliver the treatments in vivo, we delivered the treatments from an injectable alginate gel. In vitro measurement of release kinetics demonstrated that the release of tmSCF slightly was faster than for tmSCFPLs or tmSCFNDs (Supplementary Fig. 7). The gel was completely degraded after seven day. To examine whether tmSCF-based treatments could enhance revascularization, we ligated the femoral artery of male mice and implanted treatments encapsulated in alginate beads at the ischemic site. Blood flow recovery was monitored using laser speckle imaging for 14 days. We found that there was significantly higher relative blood flow recovery in tmSCFPL/ND groups in comparison to the alginate control group (Fig. 3A, B). Immunohistochemical staining for PECAM-1 and α-smooth muscle actin (α-SMA) revealed that there was a significant increase in small blood vessels formed at Day 14 in the tmSCFND treatment group in the thigh muscles while there were increased large blood vessels in mice treated with tmSCFPLs or tmSCFNDs in both the calf and thigh muscles (Fig. 3C–E; Supplementary Fig. 8).

### Diabetic patients have reduced endothelial stem cell factor expression

Our group and others have identified mechanisms of therapeutic resistance to growth factors^35,36,40–43^, including the loss of cell surface proteoglycans^35,36,40–43^ and expression of angiogenesis inhibiting growth factors^44^. Diabetic patients in particular have reduced recruitment of stem cells to ischemic regions, circulating EPCs, and endothelial cell function^45,46^. We examined the expression of SCF/tmSCF in skin samples from diabetic and non-diabetic patients to assess differences. We found a marked reduction of SCF expression in endothelial cells in the skin of type 2 diabetic (T2D) patients (Supplementary Fig. 9A, B). The expression of c-Kit in endothelial cells was higher in patients with T2D in comparison to non-diabetic patients (Supplementary Fig. 9C, D). Conversely, SCF levels were similar between the groups in skin fibroblasts (Supplementary Fig. 9E, F). In addition, we found an increase in c-Kit in mast cells in the skin from diabetic patients (Supplementary Fig. 9G, H). These results support that there is a significant alteration in SCF and c-Kit in the skin of diabetic patients that may contribute to altered recovery of ischemia.

### Transmembrane stem cell factor proteoliposomes and nanodiscs enhance revascularization in ob/ob mice

**Fig. 4:**
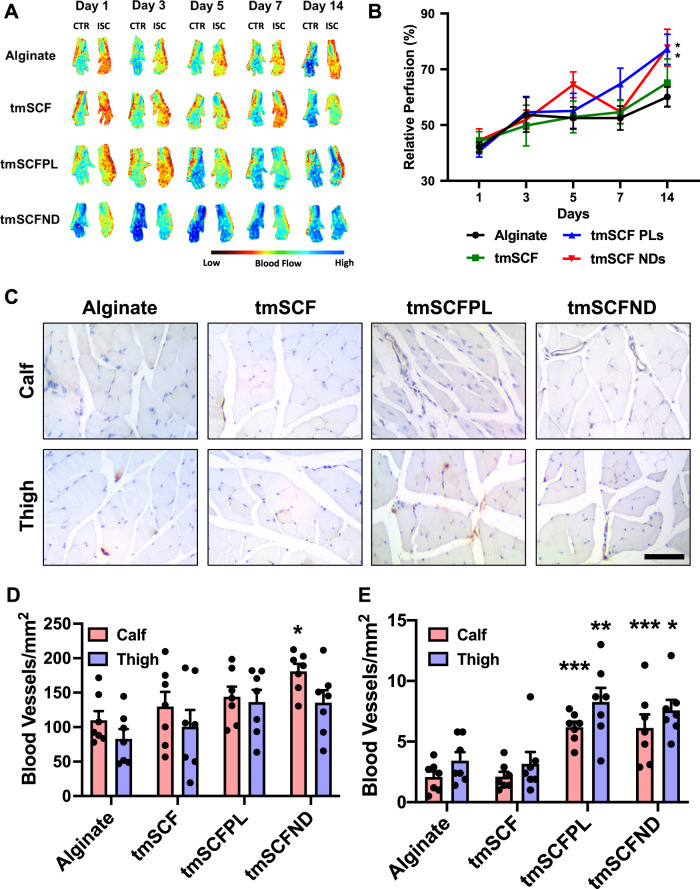
Evaluation of treatments in a hindlimb ischemia model on ob/ob mice. **A** Representative mice foot images taken by laser speckle imaging. **B** Relative blood flow recovery after hind limb ischemia surgery on ob/ob mice (*n* = 11 for tmSCF, 12 for other groups). *p* = 0.0424 on tmSCFPL vs control, and *p* = 0.0495 on tmSCFND vs control. Kruskal–Wallis test with Dunn’s post hoc was used. **C** Immunostaining for PECAM on mice on the muscles from the ischemic calf and thigh muscle of the mice after 14 days. Scale bar = 100 µm. **D** Quantification small blood vessels in the calf and thigh muscle counted from PECAM immunostaining images (*n* = 7). *p* = 0.0116 on tmSCFND vs alginate. **E** The number of large blood vessels in the tissues (*n* = 7). *p* = 0.008 on tmSCFPL vs control and *p* = 0.009 on tmSCND vs control on calf muscle. *p* = 0.0035 on tmSCFPL vs control and *p* = 0.0122 on tmSCFND vs control on thigh muscle. Two-sided one-way ANOVA with Dunnett’s post hoc test was used. * indicates *p* value < 0.05, ** < 0.01, and *** <0.001. All the error bars are SEM.

To examine the effect of the treatments under diabetic conditions, we repeated the hind limb ischemia model in female ob/ob mice. After 14 days, there was significantly higher blood flow recovery in tmSCFPLs and tmSCFNDs treated groups in comparison to the alginate control-treated mice (Fig. 4A, B). Immunohistochemical staining on ob/ob mice to detect endothelial cells (PECAM-1) and smooth muscle cells (α-SMA) showed that there were increased small blood vessels under tmSCFNDs treatment in the calf muscle while there were increased numbers of mature blood vessels in the groups treated with tmSCFNDs or tmSCFPLs in both the calf and thigh (Fig. 4C–E; Supplementary Fig. 10). Treatment with tmSCF alone did not enhance the mature blood vessel formations in ob/ob mice in comparison to WT mice, indicating the better treatment potential with nanocarriers. Overall, tmSCF treatment with nanocarriers improved blood flow recoveries in both WT and ob/ob mice in the hind limb ischemia model.

### Transmembrane stem cell factor nanodiscs induce CD34-CD133+ endothelial progenitor cells mobilization to the peripheral blood

**Fig. 5:**
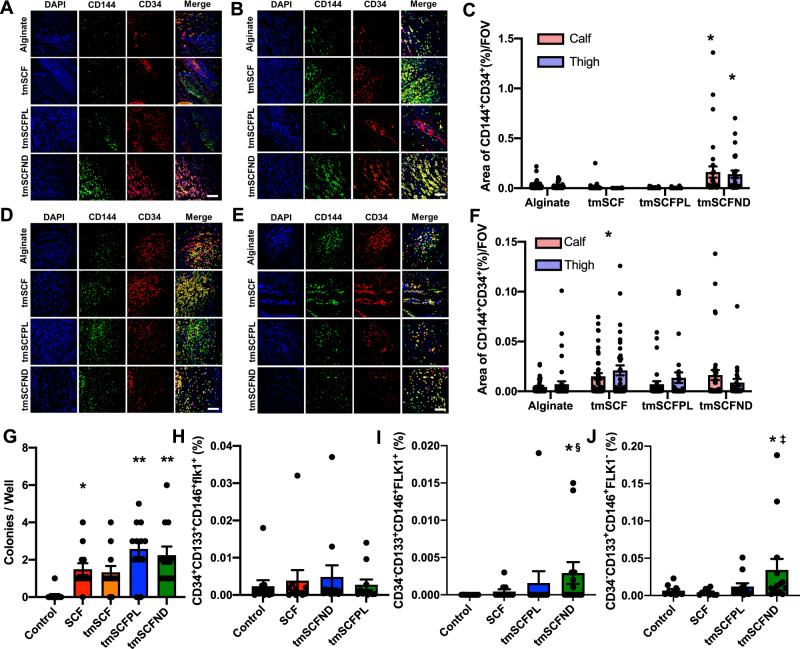
EPCs are recruited to peripheral blood and ischemic site. **A**, **B** Representative immunostaining images of CD34 (red) and CD144 (green) on WT mice calf and thigh muscle, respectively. Scale bar is 300 µm. **C** CD34 and CD144 double positive areas were quantified in WT mice (*n* = 3 for alginate and tmSCF, 4 for tmSCFPL and tmSCFND). **p < 0.01 versus control (**D**, **E**). Representative immunostaining images of CD34 (red) and CD144 (green) on ob/ob mice calf and thigh muscle, respectively. Scale bar is 300 µm. **F** CD34 and CD144 double positive areas were quantified in ob/ob mice (*n* = 3 for tmSCFPL, 4 for alginate and tmSCFND, and 5 for tmSCF). **p < 0.01 versus control. **G** Average large EPC colony number per well (*n* = 12). **H** Frequency of CD34^+^CD133^+^CD146^+^FLK1^+^ cells in peripheral blood after subcutaneous injection (*n* = 11 for alginate, SCF and tmSCFPL, and 12 for tmSCFND). **I** Frequency of CD34^−^CD133^+^CD146^+^FLK1^+^ cells in peripheral blood after subcutaneous injection (*n* = 11 for alginate, SCF and tmSCFPL, and 12 for tmSCFND). *p* = 0.0454 (tmSCFND vs control) and *p* = 0.00302 (tmSCFND vs SCF). **J** Frequency of CD34^−^CD133^+^CD146^+^FLK1^−^ cells in peripheral blood after subcutaneous injection (*n* = 10 for alginate, 11 for SCF, 12 for tmSCFPL and 14 for tmSCFND). *p* = 0.0314 (tmSCFND vs control) and *p* = 0.0031 (tmSCFND vs SCF). *, ^†^, ^‡^ and ^§^ indicate significant difference over alginate control, tmSCF, tmSCFPL, and SCF, respectively (*p* < 0.05; Kruskal–Wallis; one-way analysis of variance). All the error bars are SEM.

Our trafficking studies supported that the proteoliposomal formulation of tmSCF was preferentially taken up by endothelial cells in comparison to free tmSCF or tmSCFNDs. Consistent with these findings, treatment with tmSCFPLs induced significantly higher tube formation in endothelial cells in comparison to cells with control treatment while tmSCFNDs did not (Supplementary Fig. 5). We next treated bone marrow cells for 30 min and found that tmSCFPLs and tmSCNDs increased the population of CD34^−^CD133^+^CD146^+^ cells in comparison to the control or tmSCF treated cells (Supplementary Fig. 11A). A recent study identified that CD34^−^CD133^+^ EPCs were the most potent EPC phenotype for treating ischemia in the recent study^47^, suggesting that tmSCFPLs and tmSCFNDs specifically induced this potent EPC phenotype. We immunostained for CD34^+^/CD144^+^ in the muscles of WT mice with hind limb ischemia and found increased EPCs in the ischemic muscles of tmSCFNDs treated mice, indicating the recruitment of EPCs to the ischemic region (Fig. 5A–C). We also immunostained for EPCs in the muscles of ob/ob mice from the hind limb ischemia study and found increased CD34^+^/CD144^+^ in the ischemic muscles of tmSCF treated mice (Fig. 5D–F). We also conducted an EPC colony formation assay on BMMNCs and found that both tmSCFPLs and tmSCFNDs induced a greater number of large EPC colonies in comparison to the control (Fig. 5G). Together, these results suggest that nanocarrier formulations of tmSCF are more active in inducing bone marrow cell mobilization and EPC recruitment in comparison to the free tmSCF.

Following injury or development of ischemia, EPCs mobilize and home to the region of injury^48^. Soluble SCF is known to participate in both the mobilization and homing of EPCs in concert with other cytokines^49^. To test if the tmSCF-based treatments induced EPC mobilization, we performed subcutaneous injections of the treatments in mice for four days consecutively and then performed flow cytometry on the peripheral blood to identify EPCs. While we did not observe significant changes in the conventional EPC population of CD34^+^CD133^+^CD146^+^FLK1^+^ cells (Fig. 5H), we found that there were increases in CD34^−^CD133^+^CD146^+^FLK1^+^ and CD34^−^CD133^+^CD146^+^FLK1^−^ cells in the tmSCFND treated group (Fig. 5I, J). This result indicates that tmSCFNDs induce mobilization of the potent CD34^−^ subtype of EPCs to peripheral blood as described in previous work^47^. We also examined the EPC population in bone marrow after four consecutive days of subcutaneous injection of our treatments and found no significant alterations in the CD34^−^CD133^+^CD146^+^FLK1^+^ or CD34^+^CD133^+^CD146^+^FLK1^+^ populations in the bone marrow following the treatments (Supplementary Fig. 11B, C).

To further confirm the ability of the treatments to induce EPCs, we injected the mice for four consecutive days with the treatments and then isolated the bone marrow from the mice. We analyzed the bone marrow using flow cytometry with staining for CD45, CD115, CD34, CD146, CD133, and VEGFR2. We found that there was a significant increase in cells with a CD45−/CD115−/CD34+/CD133−/VEGFR2+ phenotype in both the adherent and non-adherent cell populations (Supplementary Fig. 12). In addition, we isolated cells from the bone marrow after three days of injection with the treatment and then grew the cells in culture for seven days. We measured the acetylated LDL uptake in the cells and found increased numbers of cells LDL uptake in the cells isolated from mice treated with SCF, tmSCFNDs, or tmSCF PLs (Supplementary Fig. 13).

## Discussion

Overall, our study demonstrated that tmSCF is an effective therapeutic protein for enhancing revascularization in peripheral ischemia without the accompanying activation of mast cells. Membrane proteins have been traditionally viewed as targets for drug development and are often difficult to isolate and maintain in solution in the absence of the cell membrane. Our work demonstrates that the transmembrane form of SCF may provide advantages over the soluble SCF, with reduced effects on mast cells while maintaining activity to induce bone marrow cell mobilization and angiogenesis. The treatment of ischemia in diabetes has been a recurrent clinical problem, especially among diabetic patients receiving bypass surgery or percutaneous interventions^50,51^. Our work has demonstrated that there are alterations in SCF and c-Kit expression in diabetic patients and that tmSCF-based therapeutics are effective in treating ischemia in diabetic mice. Additionally, our work supported that the nature of the lipid nanocarrier used to encapsulate tmSCF can significantly alter its interactions with different cell types, leading to alterations in the regeneration process during revascularization in ischemia.

**Fig. 6:**
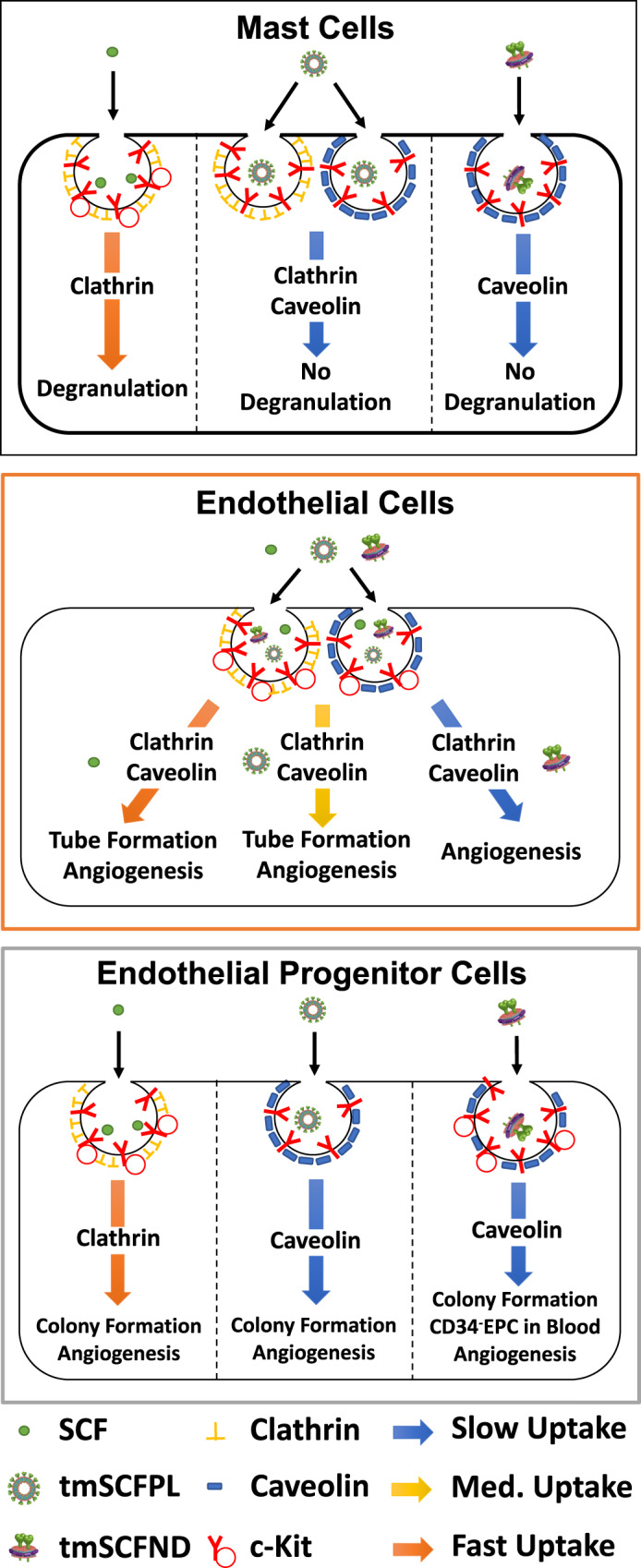
Summary of the experimental findings in the studies. Mast cells use primarily a clathrin-mediated pathway to internalize SCF, leading to mast cell activation and anaphylaxis. In contrast, mast cells use predominantly clathrin and caveolin-mediated pathway to uptake tmSCF-based treatments and these treatments do not cause mast cell activation. Endothelial cells use both of clathrin- and caveolin-mediated pathway to uptake SCF, inducing angiogenesis in endothelial cells. Endothelial cells use both of clathrin- and caveolin-mediated pathway to internalize tmSCFPLs with medium uptake speed, triggering tube formation of endothelial cells and therapeutic angiogenesis. However, tmSCFNDs are internalized through clathrin/caveolin-mediated pathways with slow kinetics and do not induce an angiogenic response from mature endothelial cells. Endothelial progenitor cells (EPCs) use clathrin-mediated pathway to uptake SCF, triggering colony formation of EPCs and bone marrow cell mobilization. For tmSCF-based treatments, EPCs use a caveolin-mediated pathway for internalization leading to colony formation and angiogenesis. Treatment with tmSCFNDs further induced the mobilization of CD34^−^CD133^+^ EPCs to the peripheral blood.

A major difference in the tmSCF-based treatments in comparison to soluble SCF was the slower uptake kinetics and cellular trophism of tmSCF for endothelial cells or EPCs depending on the nanocarrier used for delivery. A summary of the findings of our trafficking studies is shown in Fig. 6. Mast cells use predominantly a clathrin-mediated pathway to internalize SCF, clathrin and caveolin-mediated pathways for tmSCFPLs, and a caveolin-mediated pathway for tmSCFNDs. The clathrin-mediated pathway has more rapid internalization and causes increased activation of mast cells^39^. Our studies suggest that mast cell activation does not occur for tmSCF-based treatments because of the slower uptake, greater utilization of the caveolin internalization pathway, and weaker activation of the c-Kit receptor. A recent study used protein engineering techniques to create a modified version of SCF that led to partial agonism of the c-Kit receptor, allowing for therapeutic activity towards hematopoietic stem cells without mast cell activation^52^. These findings are consistent with our studies that show weaker activation of c-Kit with reduced mast cell activation and therapeutic activities.

Our studies also revealed that the type of nanocarrier used to deliver tmSCF elicited a differential response based on cell type. Endothelial cells were most responsive to tmSCF proteoliposomes, showing increased tube formation, angiogenesis, and mature vessel formation (Fig. 6). In contrast, EPCs were more responsive to tmSCF nanodiscs and induced greater colony formation and mobilization of CD34^−^CD133^+^ EPCs to the peripheral blood. Our studies suggest that endothelial cells use a combination of clathrin and caveolin-mediated pathways to internalize tmSCFPLs and tmSCFNDs while EPCs use only a caveolin-mediated pathway to internalize these treatments. Notably, the size of proteoliposomes used here was chosen as it was the optimal size for delivering other therapeutic membrane proteins to induce angiogenesis and endothelial cell activation in our previous studies^36,40,41,43^. One potential explanation for the differences in uptake mechanism between the treatments may be that the size of the therapeutic compounds. A previous study demonstrated that cells use predominantly clathrin-mediated pathways to internalize particles <200 nm in size while they use almost exclusively caveolin-mediated pathways to take up particles >500 nm^53^. In our study, the size of the tmSCFNDs was around 200 nm and the size of the tmSCFPLs was approximately 400 nm. Thus, our findings are consistent with this previous work and suggest that formulating membrane protein therapeutics spanning these ranges is an effective means for controlling the therapeutic response if there are clathrin and caveolin-based difference in the cellular response to the protein. In addition, the tmSCFPLs may be recognized by receptors that are specifically found on endothelial cells (eg. LDLR, SCARB1), which may improve their uptake in endothelial cells. While endocytosis can lead to degradation of the receptor/ligand and cause downregulation of a cellular response, it can serve as a mechanism to induce sustained and enhanced signaling. The insulin receptor has been a well-studied example of this where studies have shown clathrin-mediated endocytosis to have both positive and negative effects on insulin responsive signaling^54–57^. Thus, the differential mechanism of endocytosis, based on the nanocarrier properties, provides a potential explanation for the cell type specific response as mast cells, endothelial cells and EPCs could respond differentially based on their differences in the utilization of these pathways for uptake of the c-Kit receptor.

In summary, this study demonstrates the first use of tmSCF as a therapeutic protein for the treatment of peripheral ischemia. Our work supports that there is minimal mast cell activation by tmSCF-based therapies and that the activity can be targeted to specific cell types and internalization pathways through the use of different nanocarriers. While both nanodisc and proteoliposome-delivered tmSCF are beneficial in treating ischemia, our work supports that the proteoliposomal tmSCF may act through the stimulation of angiogenesis in endothelial cells and proliferation of bone marrow cells. Conversely, nanodisc delivered tmSCF acts primarily through the mobilization and recruitment of bone marrow cells and stimulation of a CD34^−^ EPC population. Overall, our work suggests some of the benefits of SCF treatment can be recapitulated using tmSCF therapies and these appear to have improved safety as well as tailorable activity based on the lipid nanocarrier used.

## Methods

### Preparation of tmSCF Proteoliposomes

For the production of recombinant tmSCF, HEK-293Ta cells were transduced with lentiviral vectors with constitutive expression of 6X His tagged tmSCF. Viruses were produced in HEK-293Ta cells using human lentiviral packaging system according to the manufacturer’s instructions (Genecopoeia). Puromycin was used to select only for transduced cells. The cells were lysed in a buffer containing 20 mM Tris (pH 8.0), 150 mM NaCl, 1% Triton X-100, 2 mM sodium orthovanadate, 2 mM PMSF, 50 mM NaF, and protease inhibitors (Roche) at room temperature. Transmembrane stem cell factor (tmSCF) was then isolated using cobalt chelating column (Chelating column; GE Healthcare), and the buffer was exchanged in 1X PBS. After concentrating the protein solution with Centriprep concentrators (Millipore), the final working concentration of protein was found to be 100 µg/ml using a BCA Protein Assay kit (Thermo Scientific). Purity of the protein was confirmed by SDS-PAGE followed silver staining of the gel. To prepare liposomes, stock solutions (10 mg mL^−1^) of 1,2-dioleoyl-sn-glycero-3-phosphocholine (DOPC), 1,2-dioleoyl-sn-glycero-3-phosphoethanolamine (DOPE), cholesterol, and sphingomyelin were prepared in chloroform. The lipids were mixed in the volumetric ratio 2:1:1:1 in a round bottom flask and the chloroform was removed on rotatory evaporator attached to a vacuum pump. Liposomes were resuspended in 4-(2-hydroxyethyl)-1-piperazineethanesulfonic acid buffer by vortexing, sonication, and freeze thawing. Extruding device (Avanti Polar Lipids) with polycarbonate membranes (400 nm) was used to homogenize the liposome population. A mild detergent, n-octyl-β-D-glucopyranoside (1% w/v) was added to both the liposome and the recombinant tmSCF. The protein and liposomes were then combined and the detergent was removed by timed serial dilution (every 30 min, 10% dilution up to 2 h), dialysis, and treatment with Biobeads (Biorad). The final tmSCF concentration in tmSCFPLs and tmSCFNDs were determined 55 and 77 μg/ml, respectively. Details of the compounds used in the study can be found in Supplementary Table 1.

### Preparation of tmSCF nanodiscs

As a lipid source, 1-palmitoyl-2-oleoyl-sn-glycero-3-phosphocholine (POPC) was used in this study. POPC was stored in chloroform, so we first removed chloroform by rotary evaporator. After that, POPC was resuspended in sodium cholate (100 mM). MSP protein was then added to phospholipid solution, and the detergent concentration was adjusted between 14 and 40 mM. This construct was incubated for 15 min at 4 °C. To solubilize the membrane protein, tmSCF was incubated in the n-octyl-β-D-glucopyranoside (1% w/v) for 15 min at 4 °C. After 15 min incubation of lipid construct and tmSCF protein, these were combined and incubated for 1 h at 4 °C. Final detergent concentration was adjusted to 20 mM with sodium cholate. Finally, detergent was removed by dialysis and biobeads.

### Characterization of proteoliposome and nanodisc

The size and zeta potential of the proteoliposomes and nanodiscs were characterized by dynamic light scattering (Malvern Zetasizer Nano ZS). Calibration was performed using 54 nm polystyrene particles. For TEM imaging of proteoliposomes, carbon support grids (300 mesh Cu; EM Sciences) were treated with glow discharge at 50 mA for 2 min (Emitech K100X; Quorum Technologies). The samples were then applied to the grids and the excess liquid removed with a filter paper. 2% uranyl acetate solution was used to stain glids, and images were taken using an FEI Tecnai Transmission Electron Microscope (TEM). For cryo-electron microscopy imaging, the liposomes were plunge-frozen in liquid ethane on carbon holey film grids (R2X2 Quantifoil; Micro Tools GmbH, Jena, Germany). The grids were transferred to a cryo-specimen holder (Gatan 626) under liquid nitrogen and put in a microscope (JEOL 2100 LaB6, 200 keV), Grids were maintained at close to liquid nitrogen temperature during EM session (−172 to −180 °C).

### tmSCF protein release kinetics from alginate beads

Purified tmSCF protein was first labeled with Alexa Fluor™ 488 Microscale Protein Labeling Kit (Thermo Scientific), then tmSCFPLs and tmSCFNDs were fabricated using this labeled protein. Equal volume of treatments and 4% sodium alginate solution were mixed, then the solution was extruded from 30G needle into a 1.1% calcium chloride solution to crosslink for 1 h. Protein amount was calibrated based on the protein fluorescent signal. Fabricated alginate beads were incubated in the 5 ml of 1% BSAPBS in plastic scintillation vial and incubated at 37 °C. 100 µl of released protein was collected each time point and fluorescent intensity was measured by plate reader. The same amount of 1% BSAPBS was replaced each time point to compensate.

### Cell lines and cell culture

Human umbilical cord endothelial cells (Promocell or Thermofisher) from a single donor were cultured in MCDB-131 culture medium with 7.5% fetal bovine serum (FBS), endothelial cell growth supplement (R&D Systems), L-glutamine and antibiotics. MC/9 cell were grown in high glucose DMEM with 2 mM L-glutamine, 0.05 mM 2-mercaptoethanol, 10% T-Cell Culture Supplement (354115; Corning) and 10% FBS. All cells were cultured in an incubator at 37 °C under a 5% CO_2_ atmosphere. MC9 mast cells (ATCC) were cultured in high-glucose DMEM medium supplemented with 10% fetal bovine serum, L-glutamine, and penicillin–streptomycin.

### Tube formation assay

Cells were stained with cell tracker green, cultured, and starved 24 h prior to the experiment. Growth factor reduced Matrigel (Corning) were coated on the 96 well plate, and endothelial cells were seeded on top of it with seeding density of 4 × 10^4^/well. Treatments were then added and cells incubated for 6 or 10 h. Images were taken using high throughput imaging system (Cytation 5 Cell Imaging Multi-Mode Reader; Biotek) at each time point and the number of loop was quantified using Photoshop (Adobe).

### Cell proliferation assay

Endothelial cells were passaged into a 96-well plate and cultured in low serum media with 2% FBS for 24 h. Treatments were then added to cells. After 24 h, BrdU was added to the cells, and proliferation was assessed 12 h later using a colorimetric BrdU assay (Cell Signaling, Inc.).

### Cell migration assay

Endothelial cells were stained with cell tracker green, and passaged to confluence in 96-well migration assay kit (Platypus). Treatments were added for 24 h before assay starts. A cell stopper was then removed to allow cells to migrate. Migration area and green fluorescent intensity were measured after 16 and 24 h.

### Hind limb ischemia model

All animal studies were performed with the approval of the University of Texas at Austin Institutional Animal Care and Use Committee (IACUC) and in accordance with NIH guidelines “Guide for Care and Use of Laboratory Animals” for animal care. Eight week old male *C57BL/6J* and eight week old female *B6.Cg-Lepob/J* were used in this study. Prior to surgery, alginate beads containing the treatments were fabricated by mixing equal volume of 4% sodium alginate solution and treatments. Beads were created through extruding the alginate solution through a 30G needle into a 1.1% calcium chloride solution to crosslink for 1 h. Wild-type mice and ob/ob mice (*n* = 12–13 for WT, *n* = 11–12 for ob/ob; Jackson Labs) were used in the studies. To perform the hind limb ischemia studies, mice were anesthetized with 2–3% isoflurane gas and a longitudinal incision was made in the inguinal region of the right thigh. The femoral artery was separated from the femoral vein and nerve, and then double ligated with 6-0 silk sutures at two locations, and the artery severed at each ligation. Treatments were then implanted in the pocket created by the surgery and the wound was closed with resorbable sutures (4-0 Vicryl; Ethicon, Inc.).

### Laser speckle contrast imaging

Prior to imaging, mice were anesthetized with 2–3% isoflurane gas. Diffuse laser illumination (785 nm, 90 mW; Thorlab) was used to image the feet of the mice and speckle data was collected through a CCD camera (Cohu 4910; Scion). We converted the raw speckle data to blood low map using Matlab. The intensity of the speckle was determined through measurements made in Photoshop (Adobe), which was used to calculate the relative blood flow in comparison to contralateral control limb^58^.

### Immunohistochemistry on human skin

Human skin samples were obtained from the Glasgow Caledonian University Skin Research Tissue Bank, Glasgow UK. The tissue bank has NHS research ethics approval to supply human skin for research (REC REF: 16/ES/0069). All methods were carried out in accordance with relevant guidelines and regulations. All experimental protocols were approved by the NHS East of Scotland Research Ethics Service. Informed consent was obtained from all subjects (no patients were under 18 years of age). Samples were formalin fixed and embedded in paraffin following standard procedures prior to sectioning. The sections were then deparaffinized in xylene and treated for 3 h with antigen retrieval solution (Dako) at 80 °C. The sections were cooled to room temperature and blocked with 20% fetal bovine serum for 45 min and then immunostained overnight with 1:100 dilution of primary antibody to PECAM-1 (Cell Signaling), SCF (Abcam), FSP1 (Abcam), c-Kit (Abcam), and mast cell tryptase (Abcam). Secondary staining was performed with secondary antibodies conjugates with Alexa Fluor 488 or 594 dye (Thermo Scientific). Following staining, the samples were imaged using confocal microscopy. For quantification, double-positive areas were quantified using Photoshop and ImageJ.

### Immunohistochemistry on mice

The thigh and calf muscles were formalin fixed and embedded in paraffin following standard procedures prior to sectioning. The sections were deparaffinized in xylene and treated for 3 h with antigen retrieval solution (Dako) at 80 °C. The sections were cooled to room temperature and blocked with 20% fetal bovine serum for 45 min and then immunostained overnight with 1:100 dilution of primary antibody to PECAM-1 (Cell Signaling), αSMA (Abcam), CD34 (Santa Cruz), and CD144 (Abcam). Secondary staining was performed with secondary antibodies conjugates with Alexa Fluor 488 or 594 dye (Thermo Scientific). Following staining, the samples were imaged using confocal microscopy. The number of small blood vessels were counted from on sections immune stained for PECAM-1, and the number of matured blood vessels was counted from αSMA positive area. For quantification from the PECAM-1 stained vessels, small vessels were defined as those with a diameter less than 15 µm. For the quantification of the double-positive areas of CD34 and CD144 were quantified using Photoshop and ImageJ.

### EPC colony formation assay

Bone marrow cells (BMCs) were harvested, and Bone marrow mononuclear cells (BMMNCs) were then separated by using Ficoll density centrifugation. Semisolid culture medium was prepared by mixing methylcellulose (MethoCult™ SF M3236; Stem Cell Technologies), 15% fetal bovine serum, 50 ng/ml murine vascular endothelial growth factor, 50 ng/ml murine basic fibroblast growth factor, 50 ng/ml murine insulinlike growth factor-1, 20 ng/ml murine interleukin-3, 50 ng/ml murine epidermal growth factor, and 100 ng/ml stem cell factor in IMDM. SCF is replaced with the same concentration of tmSCF, tmSCFPL, and tmSCFND respectively for each treatment group. Both of the BMCs and BMMCs are adjusted to 7 × 10^5^ cells/ml, then resuspended in the semisolid culture medium. Cell medium is added to six well cell culture plate and incubated for 7 days. The number of small and large colony was counted by two investigators who were blinded to the experimental conditions.

### EPCs induction to peripheral blood

To measure the ability of treatments to induce EPCs to peripheral blood, 240 µg/kg of SCF, tmSCF, tmSCFPLs, and tmSCFNDs were injected subcutaneously for consecutive four days. At the end of day 4, peripheral blood was collected for EPC marker analysis. As EPCs markers, FLK1, CD146, CD34, and CD133 were used for flow cytometry analysis. Then cells are stained for CD34-Alexa647 (BD Biosciences), CD133-PE (BioLegend), CD146-Alexa488 (BD Biosciences), and Flk-1-APC-Cy7 (BD Biosciences). An example of the gating strategy used for the studies is shown in Supplementary Fig. 14.

### Induction of EPCs in harvested bone marrow

BMCs were harvested freshly from mice for the experiment and cultured in DMEM high glucose media with 10% FBS. 100 ng/ml of SCF, tmSCF, tmSCFPLs, tmSCFNDs were added to the BMCs media, and cells were incubated for 30 min. Treatment was stopped by placing the cell plate on ice. The cells were stained for CD34-Alexa647 (BD Biosciences), CD133-PE (BioLegend), and CD146-Alexa488 (BD Biosciences). CD34-CD133+CD146+ cells are quantified by FlowJo software. Gating was performed similarly to the analysis of peripheral blood.

### Evans Blue extravasation assay

Male C57BL/6J wild-type mice were anesthetized with 2 % isoflurane and initial ear thickness was measured using Mitsutoyo Micrometer (Uline). 250 mL sterile-filtered 1% Evans blue (Sigma) in PBS was injected into the retro-orbital vein and allowed to recover. After 15 min later, mice were anesthetized again and intradermally injected into the ear pinnae with 25 μL of PBS alone (into the left ear pinna) or containing 50 mg/mL SCF, tmSCF, tmSCFPLs, or tmSCFNDs (into the right ear pinna) using a 1 mL syringe equipped with a 30G × 1/2 needle. Two hours after injections, mice were anesthetized again and ear pinnae thickness was measured and photographs were taken. Three hours after injections, mice were euthanized, ear pinnae were harvested. Ear pinnae was incubated in 300 mL dimethyl sulfoxide (DMSO) in a 48-well tissue culture plate for 20 h on a shaker at room temperature. Evans blue containing supernatant was collected, transferred into 96-well flat-bottom plate, and absorbance at 620 nm wavelength was measured using a plate reader.

### Body temperature measurement after intravenous injection on mice

Eight week old female C57BL/6J wild-type mice were anesthetized with 2% isoflurane and initial body temperature was measure by rectal probe. Treatment (120 µg/kg) was injected intravenously from tail vein and body temperature changes were measured every 10 min for 120 min (*n* = 3).

### Toxicology test

Eight week old male C57BL/6J wild-type mice were anesthetized with 2% isoflurane and initial body temperature was measure by rectal probe. Treatments (400 µg/kg) were injected intravenously at tail vein. Changes in body weight, body temperature, appearance, and activity were recorded for one week. Mice were sacrificed after one week, and liver and kidney were harvested. Tissues were embedded in paraffin, sectioned, stained with H&E, and examined for any toxicological effect.

### C-Kit internalization by flow cytometry

MC9 mast cells, bone marrow cells, and HUVECs were starved overnight with 0.5% serum prior to the experiment. The cells were treated with Brefeldin A (5 µg/ml; Cayman Chemicals, Inc.) for 2 h to inhibit a repopulation of surface c-Kit. The cells were washed with 37 °C HEPEs buffer (10 mM HEPES, 137 mM NaCl, 2.7 mM KCl, 0.4 mM Na2HPO4⋅7H2O, 5.6 mM glucose, 1.8 mM CaCl2⋅2H2O, and 1.3 mM MgSO4⋅7H2O with 0.04% BSA) and resuspended at 5 × 10^5^ cells/ml in HEPES buffer without BFA. The cells were then treated with either of SCF, tmSCF, tmSCFPLs, and tmSCFNDs (100 ng/ml) for 5, 10, 20, and 30 min. After the treatment, cells were plunged into ice to stop the c-Kit internalization. For HUVECs, accutase was used to detach cells. The surface c-Kit was stained with PE-Cy™7 anti-Mouse CD117 antibody (BD Biosciences) or PE-Cy7 anti-Human CD117 antibody (Biolegend) then surface c-Kit intensity was read by flow cytometry. The surface c-Kit intensity was normalized to initial time point (0 min). An example of the gating strategy used for the studies is shown in Supplementary Fig. 15.

### EPCs induction into the peripheral blood

To measure the ability of treatments to induce EPCs proliferation in bone marrow, 240 µg/kg of SCF, tmSCF, tmSCFPLs, and tmSCFNDs were injected subcutaneously for 4 consecutive days. On day 5, bone marrow was collected from the tibias and femurs of each mouse. Gradient density centrifugation with Ficoll Paque Plus (Sigma) was performed to isolate mononuclear cells from the bone marrow. Cell suspension was washed and resuspended in low glucose MCDB131 media with 7.5% FBS, and EGM Endothelial Cell growth medium singlequots (Lonza).

### Acetylated low-density lipoprotein uptake assay

Freshly harvested bone marrow mononuclear cells were seeded onto polymer coverslip-bottom chamber slides (Ibidi) coated with fibronectin (Sigma-Aldrich). After two days, nonadherent cells were removed with a PBS wash. Media was changed and cells were cultured for a total of seven days. On day six of culture, media was changed to endothelial growth media with 1.5% FBS to serum-starve the cells. On day seven of culture, cells were incubated with 10 mg/L of Dil-Ac-LDL (Cell Applications) for 4 h at 37 °C. Cells were washed and fixed with 4% paraformaldehyde, and counter-stained with DAPI. Cells were imaged with confocal microscopy and images were analyzed with FIJI to determine the number of cells with incorporation of DiI-labeled Ac-LDL.

### Bone marrow MNC flow cytometry characterization

Freshly harvested bone marrow mononuclear cells were seeded onto standard culture plates coated with fibronectin (Sigma-Aldrich) in endothelial growth media with 7.5% FBS. After two days, nonadherent cells were removed with a PBS wash and seeded onto separate culture plates coated with fibronectin. Media was changed and cells were cultured for a total of seven days. On day seven of culture, adherent cells were detached with Accutase (Sigma-Aldrich) and labeled with CD34-AF647, CD133-PE, VEGFR2, FLK1-APC/Cy7, CD146-AF488, CD31-BV421, CD115-BB700, CD45-AF594 (BD Biosciences) antibodies (Supplementary Table 2). The cells were incubated in the antibodies at 10 μg/ml for 30 min at 4 °C. After incubation with the antibodies, the detached cells were centrifuged and the supernatant was removed. Fixing and permeabilizing buffer (BD Biosciences) was added while the cells were vortexed and incubated for 40 min. Next, the samples were centrifuged and the supernatant was removed. Following fixation, cells were centrifuged with washing buffer two more times, before they were resuspended in stain buffer (BD Biosciences) and measured. A BD LSR II Fortessa Flow Cytometer (BD Biosciences) was used to measure population fluorescent signals. At least 50,000 events were recorded per sample, and further gating and quantification was performed with FlowJo software. An example of the gating strategy used for the studies is shown in Supplementary Fig. 16.

### Clathrin, caveolin, and c-Kit colocalization assay

Prior to the experiment, bone marrow cells were harvested from 10 weeks old female C57BL/6J wild-type mice and mononuclear cells are isolated by Ficoll density centrifugation. These bone marrow mononuclear cells are cultured for seven days under endothelial media to induce spindle-shaped adherent EPCs. The EPCs, MC9 mast cells, and HUVECs were starved overnight with 0.5% serum prior to the experiment. 100 ng/ml of SCF, tmSCF, tmSCFPLs, and tmSCFNDs were then treated for 2 min and immediately plunged into ice to stop the internalization. After washing with PBS, and fixing with 4% PFA, immunostaining with clathrin (Abcam), caveolin (Abcam) and c-Kit(Invitrogen, NOVUS Biological) were conducted. Images of 30 cells are taken by confocal microscopy and we analyzed the colocalization of clathrin and c-Kit and caveolin and c-Kit by coloc 2 in Image J. Pearson’s *R* value was used for analysis.

### Immunostaining

Prior to the experiment, the cells were starved overnight with 0.5% serum prior to the experiment. The cells were then treated with 100 ng/ml of SCF, tmSCF, tmSCFPLs, or tmSCFNDs for 5 min and immediately plunged into ice to stop the phosphorylation. After washing with PBS, and fixing with 4% PFA, immunostained for phospho-c-Kit (R&D) was conducted. Images of 30 cells are taken by confocal microscopy and we analyzed the mean intensity of phospho-c-kit inside of a cell.

### Statistical analysis

All results are shown as mean ± standard error of the mean. Comparisons between only two groups were performed using a two-tailed Student’s t-test. Differences were considered significant at *p* < 0.05. Multiple comparisons between groups were analyzed by two-way ANOVA followed by a Tukey post hoc test. A two-tailed probability value <0.05 was considered statistically significant. Measurements were taken from distinct samples for each *n* for all experiments. All error bars are standard error of the mean unless otherwise noted.

## Supplementary information

Supplemental Info

## Data Availability

The data generated in this study are provided in the Supplementary Information/Source Data file. Source data are provided with this paper.

## Source data

Source Data

## References

[1] Carter PJ (2011). Introduction to current and future protein therapeutics: A protein engineering perspective. Exp. Cell Res..

[2] Walsh G (2010). Biopharmaceutical benchmarks 2010. Nat. Biotechnol..

[3] Goeddel DV (1979). Expression in Escherichia-Coli of chemically synthesized genes for human insulin. Proc. Natl Acad. Sci. USA.

[4] Reichert JM (2010). Metrics for antibody therapeutics development. mAbs.

[5] Reichert JM (2010). Antibodies to watch in 2010. mAbs.

[6] Brown LR (2005). Commercial challenges of protein drug delivery. Expert Opin. Drug Deliv..

[7] Phillips R, Ursell T, Wiggins P, Sens P (2009). Emerging roles for lipids in shaping membrane-protein function. Nature.

[8] Hynes NE, Lane HA (2005). ERBB receptors and cancer: The complexity of targeted inhibitors. Nat. Rev. Cancer.

[9] Suzuki H (1997). A role for macrophage scavenger receptors in atherosclerosis and susceptibility to infection. Nature.

[10] Hasko G, Linden J, Cronstein B, Pacher P (2008). Adenosine receptors: therapeutic aspects for inflammatory and immune diseases. Nat. Rev. Drug Discov..

[11] Broudy VC (1997). Stem cell factor and hematopoiesis. Blood.

[12] Lennartsson J, Ronnstrand L (2012). Stem cell factor receptor/c-Kit: From basic science to clinical implications. Physiol. Rev..

[13] Ding L, Saunders TL, Enikolopov G, Morrison SJ (2012). Endothelial and perivascular cells maintain haematopoietic stem cells. Nature.

[14] Li CL, Johnson GR (1994). Stem cell factor enhances the survival but not the self-renewal of murine hematopoietic long-term repopulating cells. Blood.

[15] Gardner RV, Oliver P, Astle CM (1998). Stem cell factor improves the repopulating ability of primitive hematopoietic stem cells after sublethal irradiation (and, to a lesser extent) after bone marrow transplantation in mice. Stem Cells.

[16] Zsebo KM (1992). Radioprotection of mice by recombinant rat stem cell factor. Proc. Natl Acad. Sci. USA.

[17] Zhao LR, Piao CS, Murikinati SR, Gonzalez-Toledo ME (2013). The role of stem cell factor and granulocyte-colony stimulating factor in treatment of stroke. Recent Pat. CNS Drug Discov..

[18] Ping S (2019). Stem cell factor and granulocyte colony-stimulating factor promote brain repair and improve cognitive function through VEGF-A in a mouse model of CADASIL. Neurobiol. Dis..

[19] Bath, P. M. W., Sprigg, N. & England, T. Colony stimulating factors (including erythropoietin, granulocyte colony stimulating factor and analogues) for stroke. *Cochrane Database Syst. Rev.*10.1002/14651858.CD005207.pub4 (2013). 10.1002/14651858.CD005207.pub4 PMC11441151 23797623

[20] Galli SJ (1993). Reversible expansion of primate mast cell populations in vivo by stem cell factor. J. Clin. Invest..

[21] Taylor AM, Galli SJ, Coleman JW (1995). Stem-cell factor, the kit ligand, induces direct degranulation of rat peritoneal mast cells in vitro and in vivo: Dependence of the in vitro effect on period of culture and comparisons of stem-cell factor with other mast cell-activating agents. Immunology.

[22] Ishikawa K (2015). Stem cell factor gene transfer improves cardiac function after myocardial infarction in swine. Circ. Heart Fail.

[23] Xiang FL, Liu Y, Lu X, Jones DL, Feng Q (2014). Cardiac-specific overexpression of human stem cell factor promotes epicardial activation and arteriogenesis after myocardial infarction. Circ. Heart Fail.

[24] Xiang FL, Lu X, Liu Y, Feng Q (2013). Cardiomyocyte-specific overexpression of human stem cell factor protects against myocardial ischemia and reperfusion injury. Int. J. Cardiol..

[25] Yaniz-Galende E (2012). Stem cell factor gene transfer promotes cardiac repair after myocardial infarction via in situ recruitment and expansion of c-Kit+ cells. Circ. Res..

[26] Ohtsuka M (2004). Cytokine therapy prevents left ventricular remodeling and dysfunction after myocardial infarction through neovascularization. FASEB J..

[27] Kuhlmann MT (2006). G-CSF/SCF reduces inducible arrhythmias in the infarcted heart potentially via increased connexin43 expression and arteriogenesis. J. Exp. Med..

[28] Ayach BB (2006). Stem cell factor receptor induces progenitor and natural killer cell-mediated cardiac survival and repair after myocardial infarction. Proc. Natl Acad. Sci. USA.

[29] Kanellakis P, Slater NJ, Du XJ, Bobik A, Curtis DJ (2006). Granulocyte colony-stimulating factor and stem cell factor improve endogenous repair after myocardial infarction. Cardiovasc. Res..

[30] Dehbashi M, Kamali E, Vallian S (2017). Comparative genomics of human stem cell factor (SCF). Mol. Biol. Res. Commun..

[31] Costa JJ (1998). The therapeutic use of hematopoietic growth factors. J. Allergy Clin. Immunol..

[32] Driessen RL, Johnston HM, Nilsson SK (2003). Membrane-bound stem cell factor is a key regulator in the initial lodgment of stem cells within the endosteal marrow region. Exp. Hematol..

[33] Miyazawa K (1995). Membrane-bound steel factor induces more persistent tyrosine kinase activation and longer life span of c-Kit gene-encoded protein than its soluble form. Blood.

[34] Friel J (2002). Hierarchy of stroma-derived factors in supporting growth of stroma-dependent hemopoietic cells: Membrane-bound SCF is sufficient to confer stroma competence to epithelial cells. Growth Factors.

[35] Das S (2016). Syndesome therapeutics for enhancing diabetic wound healing. Adv. Health. Mater..

[36] Monteforte AJ (2016). Glypican-1 nanoliposomes for potentiating growth factor activity in therapeutic angiogenesis. Biomaterials.

[37] Da Silva CA, Reber L, Frossard N (2006). Stem cell factor expression, mast cells and inflammation in asthma. Fundamental Clin. Pharmacol..

[38] Ekmekcioglu, S., Kurzrock, R. & Grimm, E. A. In *The Molecular Basis of Cancer*. 4th edn. (eds Mendelsohn, J. et al.) 789–808.e784 https://www.elsevier.com/books/the-molecular-basis-of-cancer/mendelsohn/978-1-4557-4066-6 (2015).

[39] Cruse G (2015). The CD20 homologue MS4A4 directs trafficking of KIT toward clathrin-independent endocytosis pathways and thus regulates receptor signaling and recycling. Mol. Biol. Cell.

[40] Das S, Majid M, Baker AB (2016). Syndecan-4 enhances PDGF-BB activity in diabetic wound healing. Acta Biomater..

[41] Das S (2016). Syndecan-4 enhances therapeutic angiogenesis after hind limb ischemia in mice with type 2 diabetes. Adv. Health. Mater..

[42] Das S, Singh G, Baker AB (2014). Overcoming disease-induced growth factor resistance in therapeutic angiogenesis using recombinant co-receptors delivered by a liposomal system. Biomaterials.

[43] Jang E, Albadawi H, Watkins MT, Edelman ER, Baker AB (2012). Syndecan-4 proteoliposomes enhance fibroblast growth factor-2 (FGF-2)-induced proliferation, migration, and neovascularization of ischemic muscle. Proc. Natl Acad. Sci. USA.

[44] Schiekofer S, Galasso G, Sato K, Kraus BJ, Walsh K (2005). Impaired revascularization in a mouse model of type 2 diabetes is associated with dysregulation of a complex angiogenic-regulatory network. Arterioscler. Thromb. Vasc. Biol..

[45] Rodrigues M (2015). Progenitor cell dysfunctions underlie some diabetic complications. Am. J. Pathol..

[46] Kang L (2009). Decreased mobilization of endothelial progenitor cells contributes to impaired neovascularization in diabetes. Clin. Exp. Pharmacol. Physiol..

[47] Friedrich Erik B, Walenta K, Scharlau J, Nickenig G, Werner N (2006). CD34−/CD133+/VEGFR-2+ endothelial progenitor cell subpopulation with potent vasoregenerative capacities. Circulation Res..

[48] Mihail Hristov AZ, Elisa ALiehn, Weber Christian (2007). Regulation of endothelial progenitor cell homing after arterial injury. Thrombosis Haemost..

[49] Li X (2017). Administration of signalling molecules dictates stem cell homing for in situ regeneration. J. Cell Mol. Med..

[50] Fitridge, R., Pena, G. & Mills, J. L. The patient presenting with chronic limb-threatening ischaemia. Does diabetes influence presentation, limb outcomes, and survival? *Diabetes Metab. Res. Rev*. 10.1002/dmrr.3242 (2019). 10.1002/dmrr.3242 31867854

[51] Takahara M (2019). Diabetes mellitus and other cardiovascular risk factors in lower-extremity peripheral artery disease versus coronary artery disease: an analysis of 1,121,359 cases from the nationwide databases. Cardiovasc. Diabetol..

[52] Ho CCM (2017). Decoupling the functional pleiotropy of stem cell factor by tuning c-kit signaling. Cell.

[53] Rejman J, Oberle V, Zuhorn IS, Hoekstra D (2004). Size-dependent internalization of particles via the pathways of clathrin- and caveolae-mediated endocytosis. Biochem. J..

[54] Ceresa BP, Kao AW, Santeler SR, Pessin JE (1998). Inhibition of clathrin-mediated endocytosis selectively attenuates specific insulin receptor signal transduction pathways. Mol. Cell Biol..

[55] Hamer I (2002). An arginine to cysteine(252) mutation in insulin receptors from a patient with severe insulin resistance inhibits receptor internalisation but preserves signalling events. Diabetologia.

[56] Bevan AP (1997). Chloroquine extends the lifetime of the activated insulin receptor complex in endosomes. J. Biol. Chem..

[57] Wang B, Balba Y, Knutson VP (1996). Insulin-induced in situ phosphorylation of the insulin receptor located in the plasma membrane versus endosomes. Biochem. Biophys. Res. Commun..

[58] Dunn AK, Bolay H, Moskowitz MA, Boas DA (2001). Dynamic imaging of cerebral blood flow using laser speckle. J. Cereb. Blood Flow. Metab..

