# Supplementary material for: Transmembrane stem cell factor protein therapeutics enhance revascularization in ischemia without mast cell activation
Source: Nat Commun. 2022 May 6;13:2497. doi: 10.1038/s41467-022-30103-2 (PMC9076913; doi:10.1038/s41467-022-30103-2)
Supplement: Supplementary file 1 — Supplemental Info [file 41467_2022_30103_MOESM1_ESM.pdf]

Supplemental Figures

Supplemental Figure 1

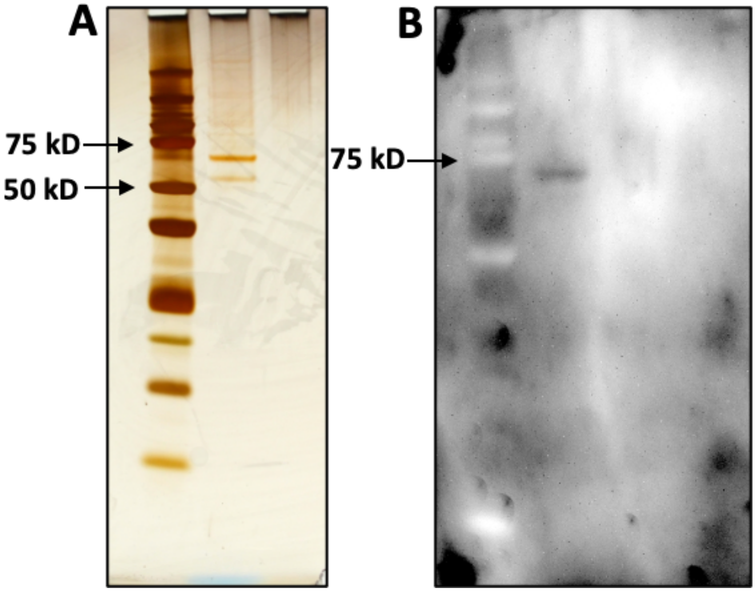

Supplemental Figure 1. Transmembrane SCF protein purification. (A) Silver staining result of purified tmSCF protein. (B) Western Blot result of purified tmSCF protein, showing its corresponding band. (Experiment repeated 3 times with similar results).

Supplemental Figure 2

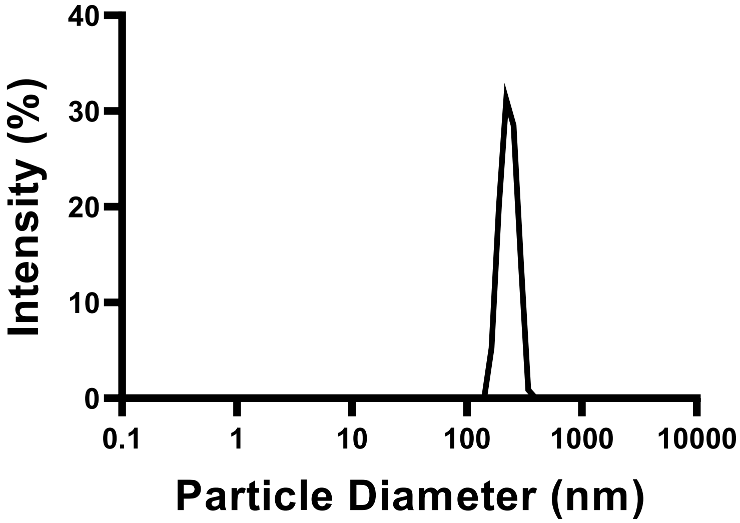

Supplemental Figure 2. DLS measurement of purified tmSCF protein. Size distribution for tmSCF measured by dynamic light scattering. The average size of tmSCF was approximately 200 nm.

Supplemental Figure 3

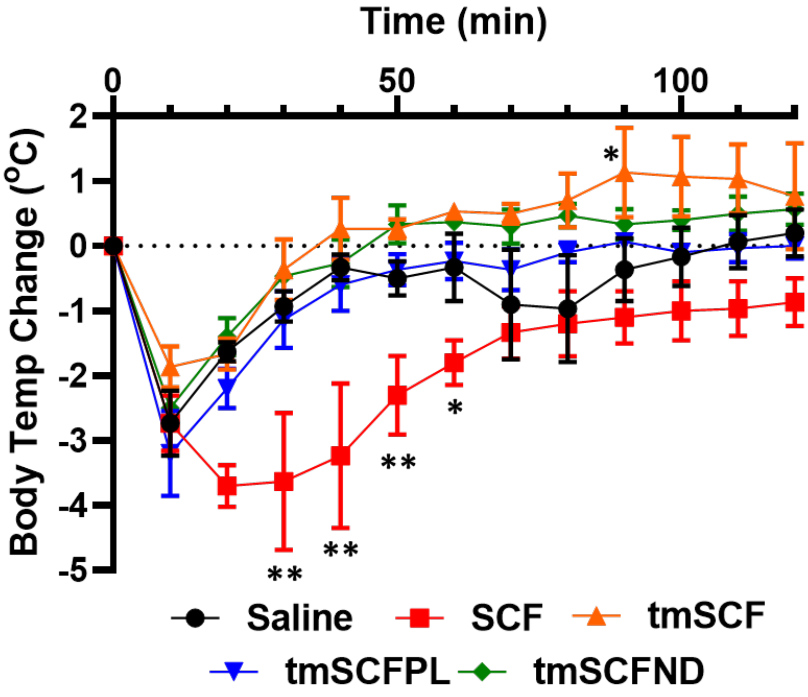

Supplemental Figure 3. Mice body temperature measurement. Treatments were injected intravenously, and body temperature changes were measured every ten minutes for two hours (n=3 biologically independent mice examined). Two-sided one-way ANOVA with Tukey's post hoc test was used. $p = 0.0028$ at 30min, $p < 0.0001$ at 40 and 50min, $p = 0.0115$ at 60min for SCF vs PBS, and $p = 0.0218$ (tmSCF vs PBS at 90 min). * indicates $p<0.05$ and ** $p<0.001$. Error bars are SEM.

Supplemental Figure 4

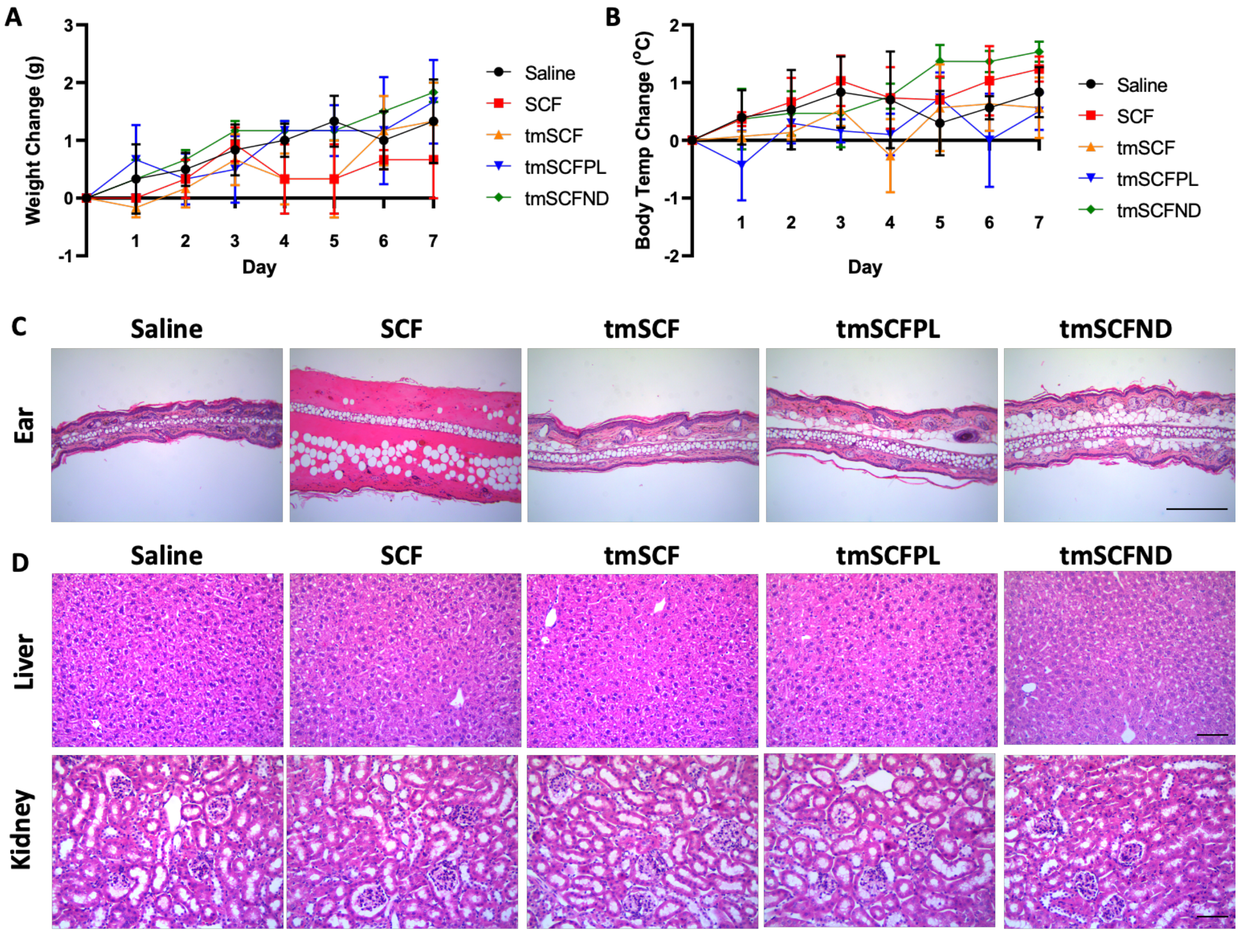

Supplemental Figure 4. Toxicology testing for the treatments. The treatments were injected intravenously at 400 μg/kg and the changes in body weight, body temperature, and appearance everyday for one week. Liver, kidney and ear tissues were harvested and stained with H&E staining. (A) Body weight changes were monitored for one week after injection (n=3 biologically independent mice examined). (B) Body temperature changes were monitored for one week after injection (n=3 biologically independent mice examined). (C) H&E staining was performed on the ear tissues one day after the intravenous injection. SCF treated mouse showed the area of edema, indicating the mast cell activation. Scale bar = 1 mm. (D) H&E staining was performed on the liver and kidney tissues seven day after the intravenous injection. Scale bar = 100 μm. Error bars are SEM.

Supplemental Figure 5

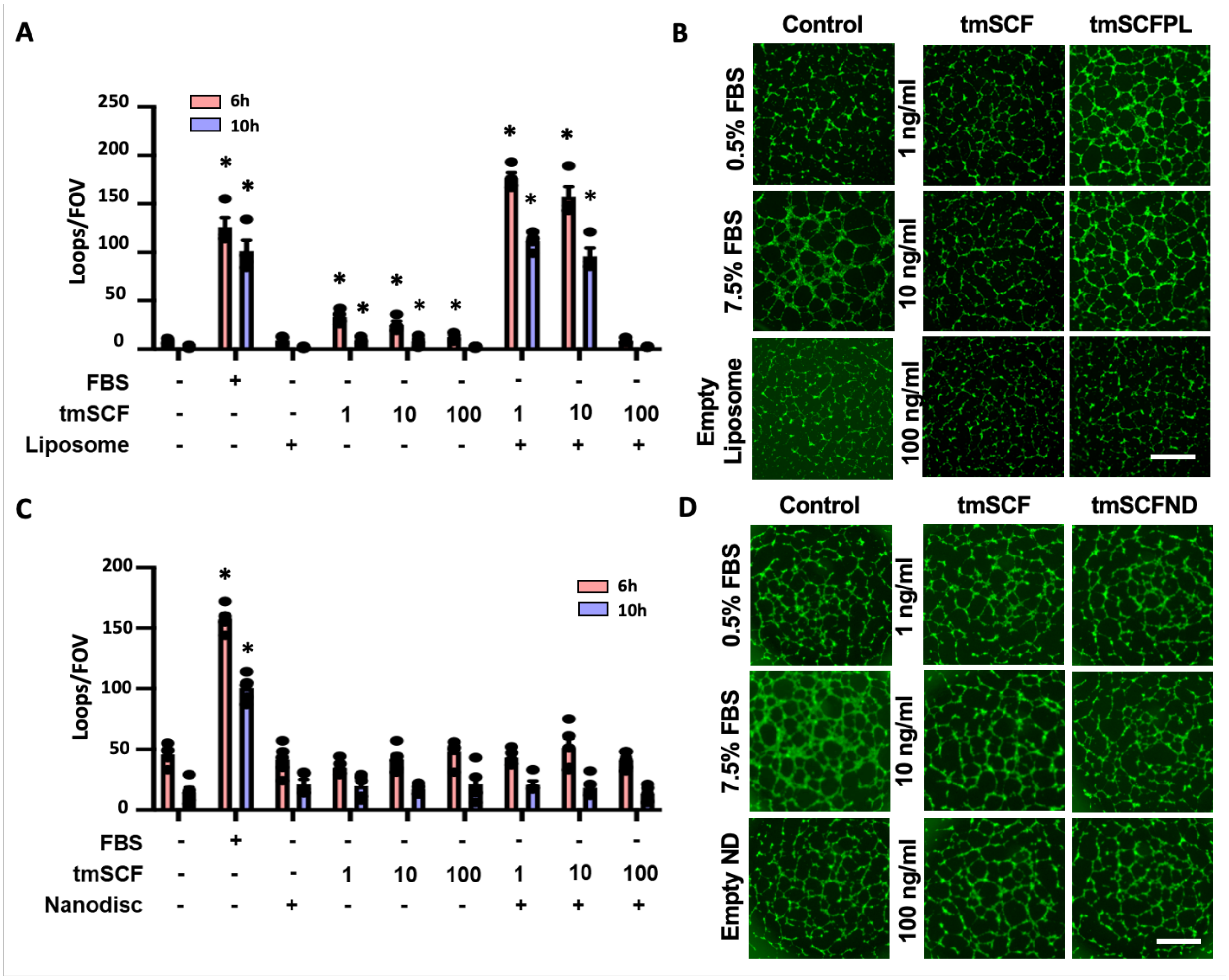

Supplemental Figure 5. Endothelial cell tube formation assay. (A) HUVECs were starved in media supplemented with 0.5% FBS for 24 hours prior to the experiment and treated with tmSCF or tmSCFPLs. Loop number was counted after 6 and 10 hours of incubation. Significantly higher number of loops were formed in 1 ng/ml and 10 ng/ml concentration of tmSCFPLs groups compared to negative control (n=4 biologically independent cells examined). Two-way ANOVA followed by a Tukey post-hoc test was used. *indicates two-tailed p value <0.05 vs control. (B) Representative images of HUVECs after 6 hours of tmSCF or tmSCFPLs treatment. Scale bar is 300 µm. (C) HUVECs were treated with tmSCF or tmSCFNDs. No significant difference was confirmed on any of the tmSCFNDs treatment group (n=4 biologically independent cells examined). Two-way ANOVA followed by a Tukey post-hoc test was used. (D) Representative images of HUVECs after 6 hours of tmSCF and tmSCFNDs treatment. Scale bar is 300 µm. Error bars are SEM.

Supplemental Figure 6

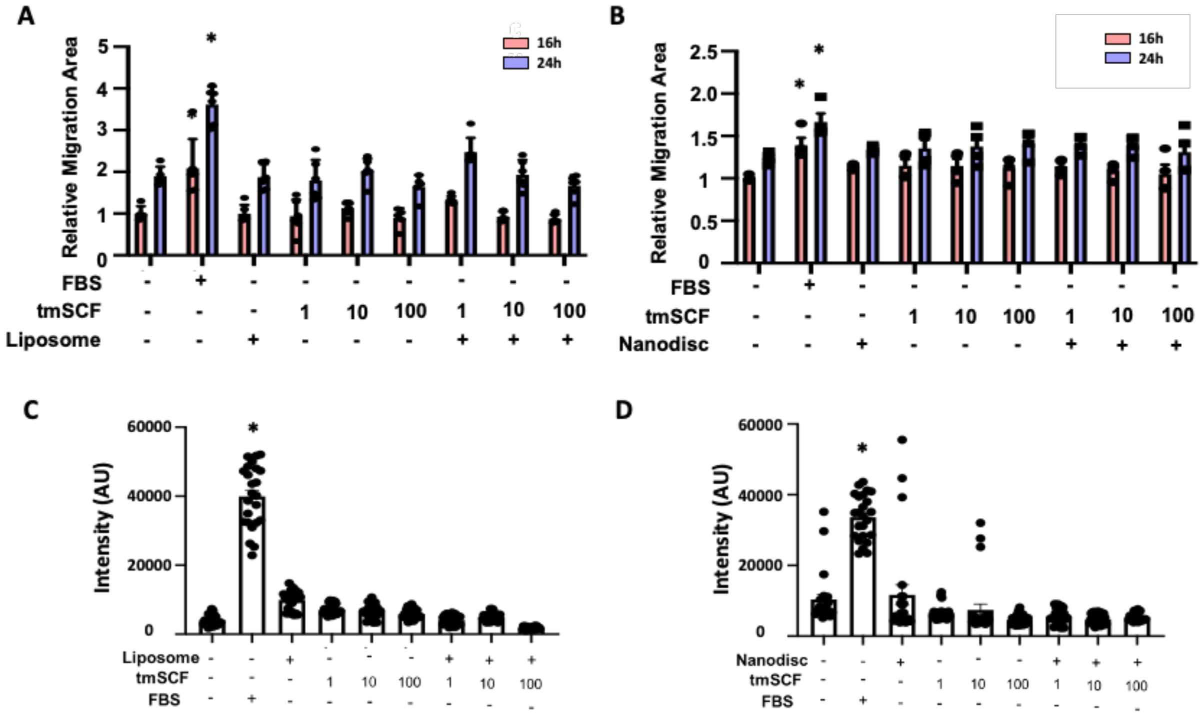

Supplemental Figure 6. Endothelial cell migration and proliferation assay. (A) Quantification results of migration assay, HUVECs were treated by tmSCFPLs. The number of the cells migrated towards the center were counted by green fluorescent signal (n=5 biologically independent cells examined). One-way ANOVA followed by a Tukey post-hoc test was used. * indicates two-tailed p value <0.05. * indicates two-tailed p value <0.05 vs control. (B) Quantification results of migration assay, HUVECs were treated by tmSCFNDs (n=4 biologically independent cells examined). The number of the cells migrated towards the center were counted by green fluorescent signal. One-way ANOVA followed by a Tukey post-hoc test was used. * indicates two-tailed p value <0.05. * indicates two-tailed p value <0.05 vs control. (C) BrdU intensity was measured after HUVECs were treated by tmSCFPLs (n=24 biologically independent cells examined over 2 independent experiments). One-way ANOVA followed by a Tukey post-hoc test was used. * indicates two-tailed p value <0.05. * indicates two-tailed p value <0.05 vs control. (D) BrdU intensity was measured after HUVECs were treated by tmSCFNDs (n=24 biologically independent cells examined over 2 independent experiments). One-way ANOVA followed by a Tukey post-hoc test was used. * indicates two-tailed p value <0.05. * indicates two-tailed p value <0.05 vs control. Error bars are SEM.

Supplemental Figure 7

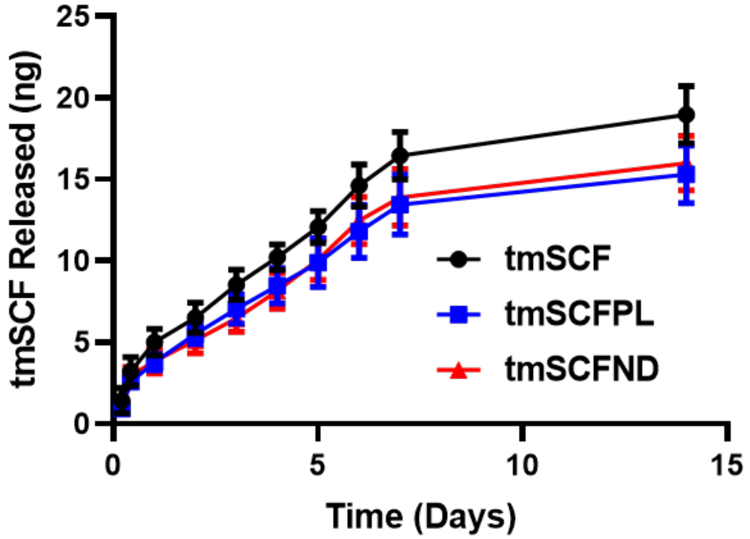

Supplemental Figure 7. Transmembrane SCF release kinetics from alginate gel showed fast release of tmSCF in comparison to tmSCFPLs or tmSCFNDs. The release kinetics of tmSCF protein, tmSCFPLs, and tmSCFNDs from alginate beads over time (n=4 independent gel examined). Error bars are SEM.

Supplemental Figure 8

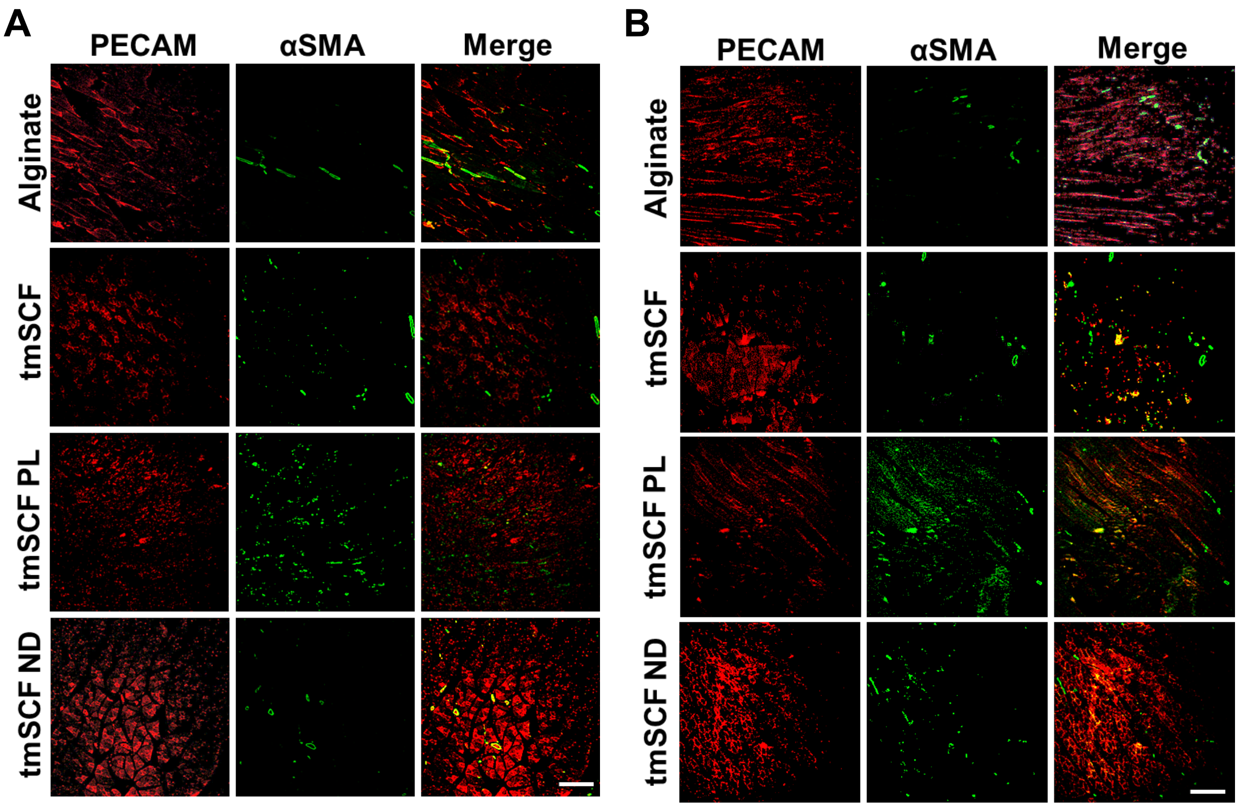

Supplemental Figure 8. Representative immunostaining images of PECAM (red) and αSMA (green) in WT mice with hind limb ischemia in the (A) calf or (B) thigh muscle (experiment repeated 4 times with similar results). Scale bar is 300 µm.

Supplemental Figure 9

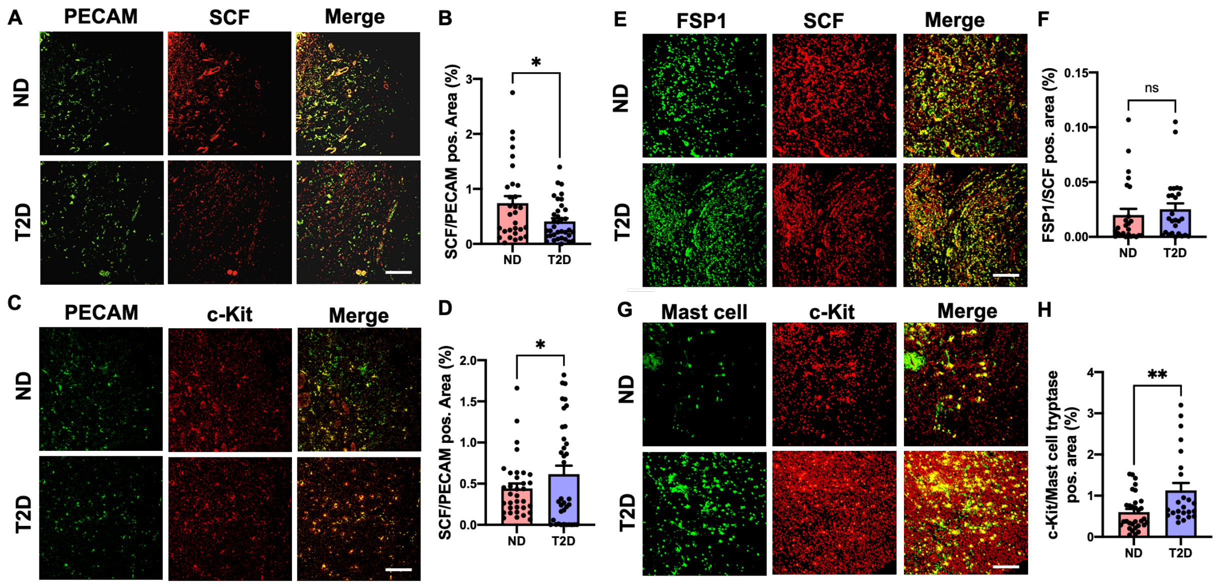

Supplemental Figure 9. Immunostaining on the skins of patients with diabetes. (A) Representative immunostaining images for PECAM and SCF. Scale bar is 300 µm. (B) Quantification of area of double positive area for PECAM and SCF. *p = 0.0146 versus nondiabetic group (n = 6). (C) Representative immunostaining images for PECAM-1 and c-Kit. Scale bar is 300 µm. (D) Quantification result of double positive area of PECAM and c-Kit. *p = 0.0478versus nondiabetic group (n = 6). (E) Representative immunostaining images of FSP1 and SCF. Scale bar is 300 µm. (F) Quantification result of double positive area of FSP1 and SCF (n = 6). p = 0.5066. (G) Representative immunostaining images of mast cell tryptase and c-Kit. Scale bar is 300 µm. (H) Quantification result of double positive area of Mast cell and c-Kit. **p = 0.005 versus nondiabetic group (n = 6). Student t test is used to determine the significance in this figure. Error bars are SEM.

Supplemental Figure 10

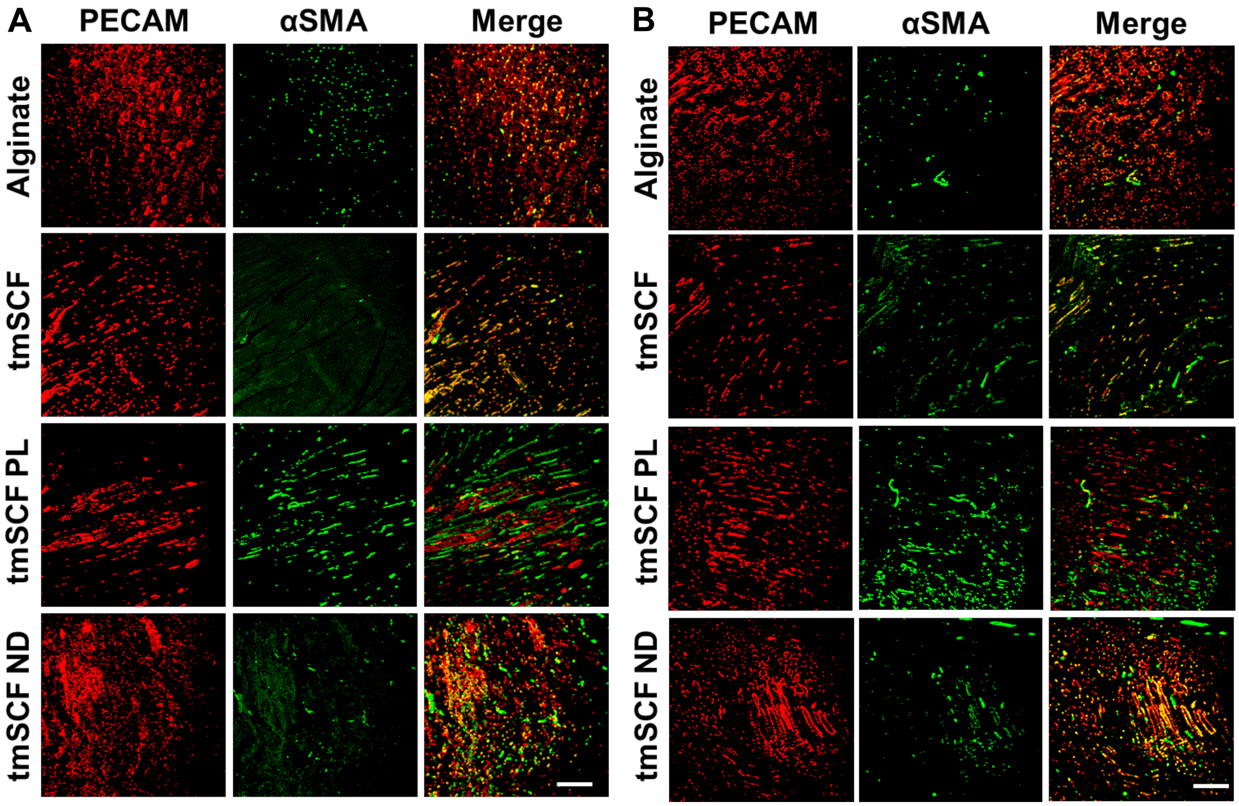

Supplemental Figure 10. Representative immunostaining images of PECAM (red) and αSMA (green) in ob/ob mice with hind limb ischemia in the (A) calf or (B) thigh muscle. Scale bar is 300 μm. (the experiment was repeated 4 times with similar results).

Supplemental Figure 11

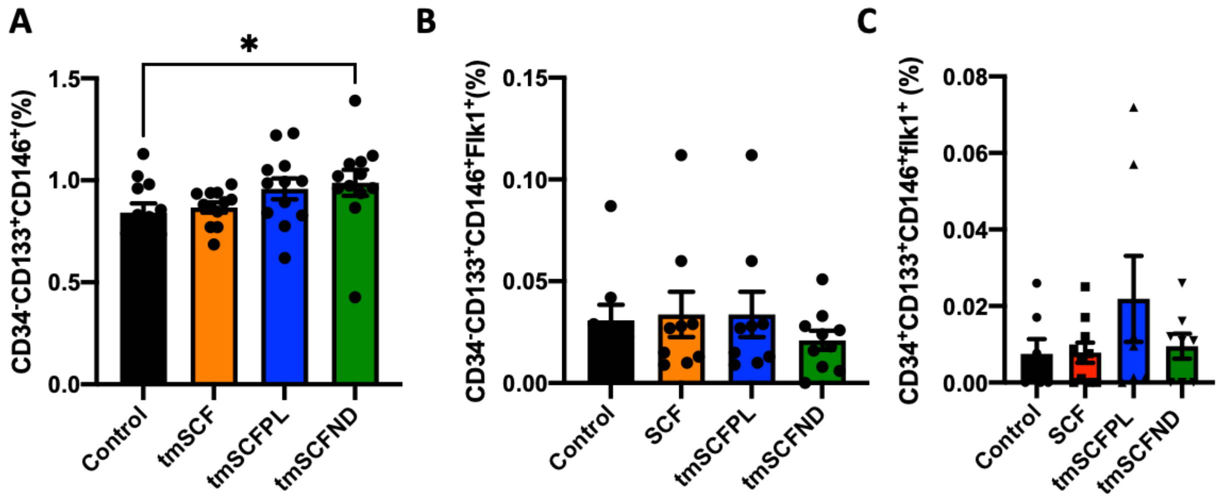

Supplemental Figure 11. EPC subpopulation analyzed by flow cytometry. (A) Bone marrow cells treated with our treatments for 30min *in vitro*, then analyzed for $CD34^{-}CD133^{+}CD146^{+}$ cells population. *p = 0.0335 versus control (n = 12 biologically independent cells examined over 2 independent experiments). Kruskal Wallis test with Dunn's post hoc was used. (B) $CD34^{-}CD133^{+}CD146^{+}FLK1^{+}$ subpopulation in bone marrow was analyzed after four days of subcutaneous injection of our treatment (n = 9 or 10 biologically independent mice examined over 3 independent experiments). (C) $CD34^{+}CD133^{+}CD146^{+}FLK1^{+}$ subpopulation in bone marrow was analyzed after four days of subcutaneous injection of our treatment (n = 7-10 biologically independent mice examined over 3 independent experiments). Error bars are SEM.

Supplemental Figure 12

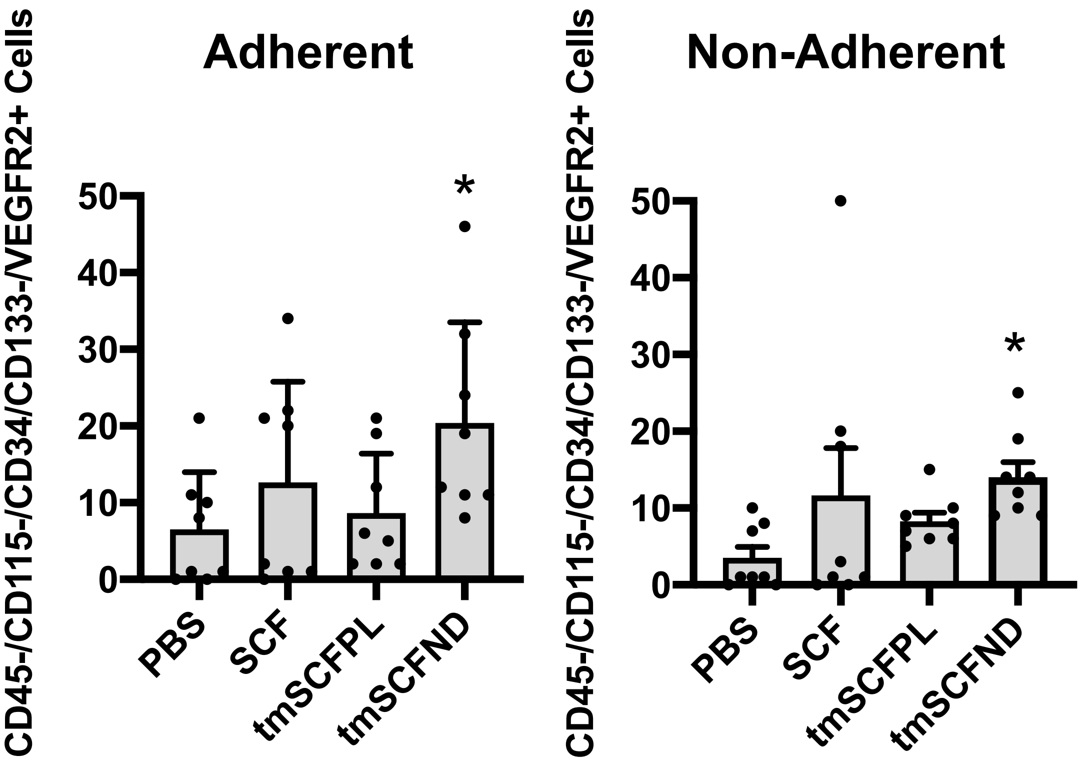

Supplemental Figure 12. Flow cytometry analysis of bone marrow in mice after daily injections of the treatments for three consecutive days. (A) CD45-/CD115-/CD34+/CD133-/VEGFR2+ phenotype in adherent cells. * p = 0.0333 tmSCFND vs control. (n = 8). (B) CD45-/CD115-/CD34+/CD133-/VEGFR2+ phenotype in non-adherent cells. * p = 0.0057 tmSCFND vs control. (n = 8). Kruskal Wallis test with Dunn's post hoc analysis was used for both adherent and non-adherent cells. Error bars are SEM.

Supplemental Figure 13

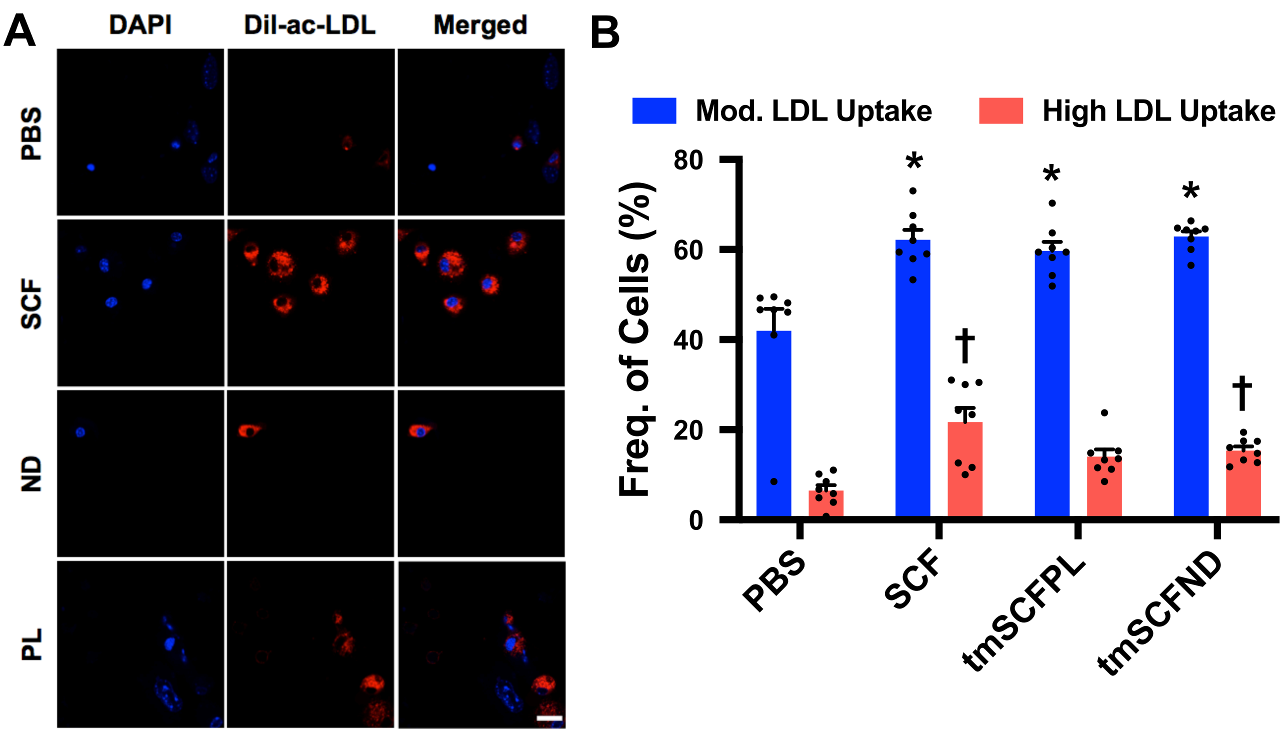

Supplemental Figure 13. Acetylated LDL uptake in cells from the bone marrow in mice after daily injections of the treatments for three consecutive days. (A) Images of cells after acetylated LDL uptake. Scale bar is 20 µm. (B) Quantification of cells with moderate (mod.) or high LDL uptake. * $p < 0.0001$ versus PBS group with moderate LDL uptake (n = 8). †p < 0.05 versus PBS group with high LDL uptake (n = 8). $p < 0.0001$ (SCF vs PBS), $p = 0.0475$ (tmSCFPL cs PBS), and $p = 0.0149$ (tmSCFND vs PBS). One-way ANOVA with Turkey post hoc analysis was used. Error bars are SEM.

Supplemental Figure 14

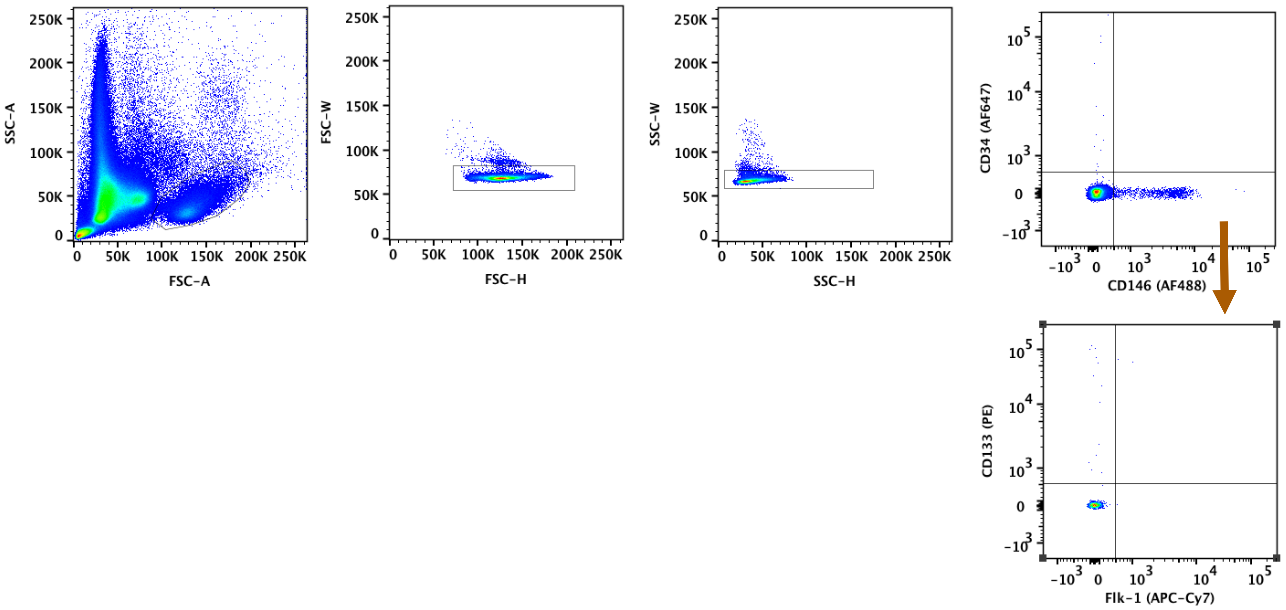

Supplemental Figure 14. Example of flow cytometry gating for analyzing EPCs in peripheral blood.

Supplemental Figure 15

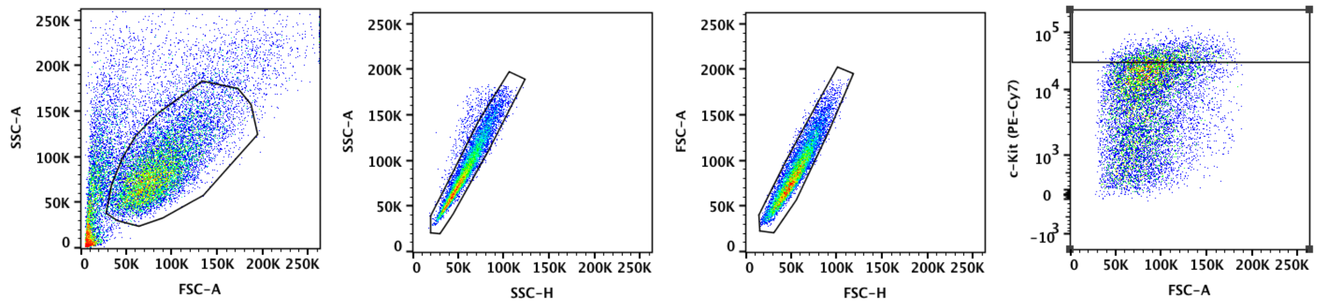

Supplemental Figure 15. Example of flow cytometry gating for analyzing c-kit internalization.

Supplemental Figure 16

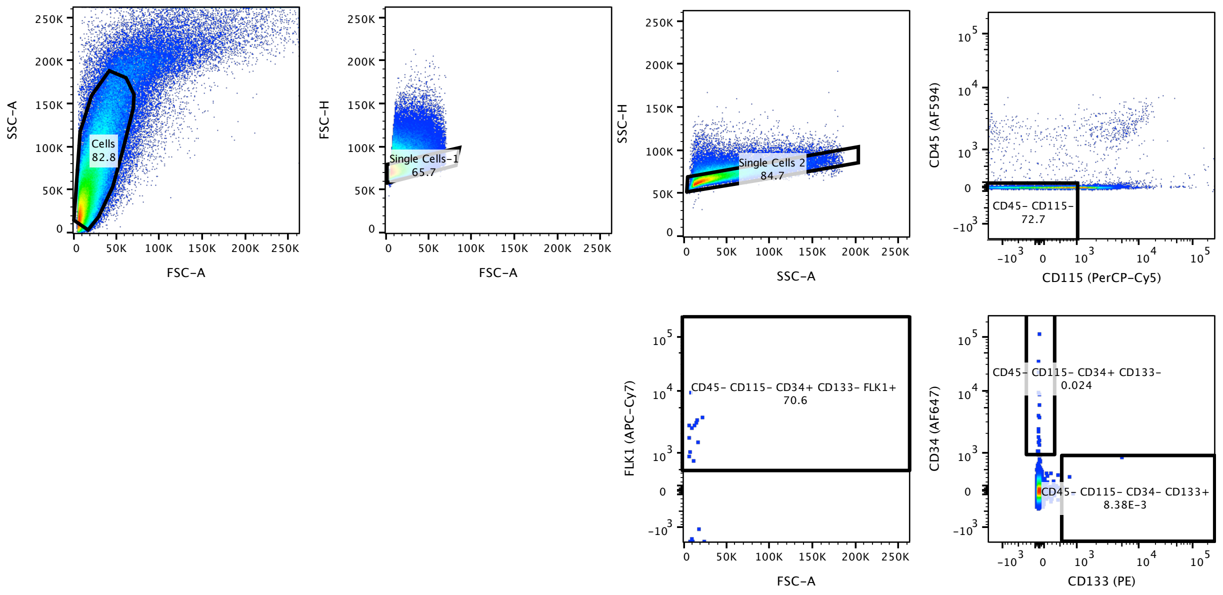

Supplemental Figure 16. Example of flow cytometry gating for analyzing EPCs in the bone marrow.

Supplemental Tables

Supplemental Table 1. *Zeta Potential Measurements*

	tmSCF	Carrier	tmSCF + Carrier
**Liposome**	-4.37 mV	-5.13 mV	-6.78 mV
**Nanodisc**		-5.80 mV	-4.85 mV

Supplemental Table 2. *Primary Antibodies Used for Immunostaining.*

Target Protein	Company	Catalog#	Species/Isotype	Dilution Ratio
GAPDH	Cell Signaling	21185	Anti-human	1:500
PECAM-1	Cell Signaling	35285	Anti-mouse	1:500
SCF	R&D system	AF-255-NA	Anti-human	1:500
c-Kit	R&D system	AF1356	Anti-human, mouse	1:500
c-Kit	Fisher Scientific	50-128-29	Anti-mouse	1:500
c-Kit	Novas Biological	NBP1-43359	Anti-mouse	1:500
FSP1	Sigma-Aldrich	07-2274	Anti-human	1:500
Mast cell Tryptase	Abcam	Ab2378	Anti-human	1:500
Clathrin	Abcam	Ab21679	Anti-mouse	1:500
Caveolin	Abcam	Ab2910	Anti-mouse	1:500
Phospho-c-Kit	Cell Signaling	33915	Anti-mouse	1:500
αSMA	Abcam	Ab21027	Anti-mouse	1:500
CD34	Santa Cruz	Sc-18917	Anti-mouse	1:500
CD144	R&D system	AF1002	Anti-mouse	1:500
Flk-1/APC-Cy7	BD Biosciences	561252	Anti-mouse	1:4
CD34/AF647	BD Biosciences	560233	Anti-mouse	1:4
CD133/PE	Biolegend	141203	Anti-mouse	1:4
CD146/AF488	BD Biosciences	562229	Anti-mouse	1:4
CD117/PE-Cy7	BD Biosciences	558163	Anti-mouse	1:1
CD117/PE-Cy7	Biolegend	313211	Anti-human	1:1
CD31/BV-421	BD Biosciences	562939	Anti-mouse	1:20
CD115/BB700	BD Biosciences	745906	Anti-mouse	1:20
CD34/AF647	BD Biosciences	560230	Anti-mouse	1:20
CD133/PE	Biolegend	141204	Anti-mouse	1:20
Flk-1/APC-Cy7	BD Biosciences	561252	Anti-mouse	1:20
CD146/AF488	BD Biosciences	562229	Anti-mouse	1:20
CD45/PE-AF594	BD Biosciences	562420	Anti-mouse	1:20

Supplemental Table 3. *Compounds Used in the Study.*

S

Compounds	Company	Catalog#
DOPC	Avanti polar lipids	850375
DOPE	Avanti polar lipids	850725
Sphingomyelin	Avanti polar lipids	860061
Cholesterol (ovine wool)	Avanti polar lipids	700000
POPC	Avanti polar lipids	850457
Octyl β-D-glucopyranoside	Sigma-Aldrich	O8001
Sodium alginate	Sigma-Aldrich	9005-38-3
Sodium cholate hydrate	Sigma-Aldrich	C6445
